# Genome-Wide Identification and Comparative Analysis of the Teosinte Branched 1/Cycloidea/Proliferating Cell Factors 1/2 Transcription Factors Related to Anti-cancer Drug Camptothecin Biosynthesis in *Ophiorrhiza pumila*

**DOI:** 10.3389/fpls.2021.746648

**Published:** 2021-10-07

**Authors:** Can Wang, Xiaolong Hao, Yao Wang, Min Shi, Zhi-Gang Zhou, Guoyin Kai

**Affiliations:** ^1^Key Laboratory of Exploration and Utilization of Aquatic Genetic Resources Conferred by Ministry of Education, Shanghai Ocean University, Shanghai, China; ^2^Laboratory for Core Technology of TCM Quality Improvement and Transformation, School of Pharmaceutical Sciences, The Third Affiliated Hospital, Zhejiang Chinese Medical University, Hangzhou, China

**Keywords:** *Ophiorrhiza pumila*, TCP transcription factors, genome-wide analysis, expression pattern, CPT biosynthesis

## Abstract

*Ophiorrhiza pumila* (*O. pumila*; *Op*) is a medicinal herbaceous plant, which can accumulate camptothecin (CPT). CPT and its derivatives are widely used as chemotherapeutic drugs for treating malignant tumors. Its biosynthesis pathway has been attracted significant attention. Teosinte branched 1/cycloidea/proliferating cell factors 1/2 (TCP) transcription factors (TFs) regulate a variety of physiological processes, while TCP TFs are involved in the regulation of CPT biosynthesis remain unclear. In this study, a systematic analysis of the TCP TFs family in *O. pumila* was performed. A total of 16 *O. pumila* TCP (*OpTCP*) genes were identified and categorized into two subgroups based on their phylogenetic relationships with those in *Arabidopsis thaliana*. Tissue-specific expression patterns revealed that nine *OpTCP* genes showed the highest expression levels in leaves, while the other seven *OpTCPs* showed a higher expression level in the stems. Co-expression, phylogeny analysis, and dual-luciferase (Dual-LUC) assay revealed that *OpTCP15* potentially plays important role in CPT and its precursor biosynthesis. In addition, the subcellular localization experiment of candidate *OpTCP* genes showed that they are all localized in the nucleus. Our study lays a foundation for further functional characterization of the candidate *OpTCP* genes involved in CPT biosynthesis regulation and provides new strategies for increasing CPT production.

## Introduction

*Ophiorrhiza pumila (O. pumila)*, belonging to the family Rubiaceae, is an important herbaceous medicinal plant and can accumulate camptothecin (CPT). CPT is a quinoline-type monoterpenoid indole alkaloid and an anticancer compound with potent DNA topoisomerase I inhibitory activity (Johnson et al., 2018). Two semi-synthetic water-soluble CPT analogs known as topotecan and irinotecan have been approved for the treatment of ovarian, colorectal, lung and cervix cancer, and HIV (Liu et al., 2015; Martino et al., 2017). Additionally, a number of other CPT derivatives have shown promising results in preclinical and clinical trials (Xie et al., 2016). Despite CPT with pharmacological relevance, its biosynthesis pathway is complex and partially deciphered (Sadre et al., 2016; Yang et al., 2021). CPT is produced by the iridoid and shikimate pathways, which supply the important precursors, such as secologanin and tryptamine. Subsequently, secologanin and tryptamine are converged into strictosidine, which is finally synthesized into CPT by some unknown catalytic enzymes (Supplementary Figure 1). At present, genomes and transcriptome data of many monoterpenoid indole alkaloid-producing plants, such as *O. pumila*, *Catharanthus roseus*, *Camptotheca acuminata*, and *Nothapodytes nimmoniana*, provided the foundation for understanding the biosynthesis and regulatory components of plant-specialized metabolites, followed by molecular characterization and functional validation of candidate genes (Kellner et al., 2015; Rather et al., 2018; Kang et al., 2021; Rai et al., 2021). At present, a few genes-encoding key enzymes, such as *OpTDC*, *OpTDC2*, *OpSTR*, *OpCPR*, *OpG10H*, *OpSLS*, *OpLAMT*, in the CPT biosynthetic pathway and several transcription factors (TFs), such as OpMYB1, OpWRKY1/2/3, and OpERF1/2/3, have been cloned and analyzed in *O. pumila* (Kai et al., 2015; Rohani et al., 2016; Udomsom et al., 2016; Shi et al., 2020; Hao et al., 2021; Yang et al., 2021; You et al., 2021). For example, OpWRKY2 acted as a positive regulator of CPT biosynthesis by directly binding and activating the gene *OpTDC* (Hao et al., 2021). Suppression of OpERF2 resulted in reducing expression of genes in the early steps that supplied a precursor for CPT biosynthesis, such as *OpTDC*, *OpG10H*, *OpSLS*, and *OpSTR* (Udomsom et al., 2016). Nevertheless, limited information is available about the regulatory mechanism of CPT biosynthesis in *O. pumila*, especially teosinte branched 1/cycloidea/proliferating cell factors 1/2 (TCP) TF.

The TCP family is a group exclusively present in the higher plants (Liu et al., 2019). The name of TCP was derived from three members of this family identified: teosinte branched 1 (TB1) in *Zea mays* (Doebley et al., 1995), cycloidea (CYC) in *Antirrhinum majus* (Luo et al., 1996), and proliferating cell factors 1/2 (PCF1/2) in *Oryza sativa* (Kosugi and Ohashi, 1997; Cubas et al., 1999). These proteins contained a highly conserved 55–59 residue-long basic helix-loop-helix (bHLH) structure at the N-terminus, known as TCP domain (Aggarwal et al., 2010). It was associated with protein nuclear localization, DNA binding, and protein–protein interaction (Cubas et al., 1999). According to the differential features in TCP domains, TCP family members were divided into two subfamilies: class I (also called PCF or TCP-P) and class II (TCP-C) (Martín-Trillo and Cubas, 2010). The most striking difference between these two subfamilies was the deletion of four-amino-acids at the N-terminal, which was exclusive in class I. Class II TCP proteins were further subdivided into the cincinnata (CIN) and cycloidea (CYC)/TB1 subclasses based on the alterations in several amino acids (Howarth and Donoghue, 2006; Horn et al., 2015). Besides the TCP domain, some members within class II had the R domain (18–20 residue arginine-rich motifs) and glutamic acid-cysteine-glutamic acid (ECE) motif (glutamic acid-cysteine-glutamic acid stretch) (Lupas et al., 1991).

Teosinte branched 1/cycloidea/proliferating cell factors 1/2 proteins were involved in diverse physiological and biological processes, such as phytohormone biosynthesis, transport, and signal transduction, leaf development, branching, embryonic growth, floral organ morphogenesis, pollen development, germination, senescence, circadian clock, and cell cycle regulation (Danisman et al., 2012; Manassero et al., 2013). Class I TCP genes, *A. thaliana* TCP (*AtTCP*)*14* and *AtTCP15*, played a vital role in seed germination and promoted embryo growth via the gibberellin signaling pathway (Resentini et al., 2015). For CIN subclass *TCP* genes, repression of *AtTCP3*, *AtTCP4*, and *AtTCP24* could disturb leaf development (Palatnik et al., 2003; Nag et al., 2009; Sarvepalli and Nath, 2011). Overexpression of CYC-like homolog GhCYC2 caused disk flowers to obtain characteristics typical for ray flowers in *Gerbera hybrida* (Broholm et al., 2008). Furthermore, several emerging lines of evidence revealed the multi-faceted role of TCP protein in plant specialized metabolism. For example, AtTCP3 interacted with R2R3-MYB proteins to stimulate flavonoid production in *Arabidopsis thaliana*, while AtTCP15 repressed anthocyanin biosynthesis when the plants were under high light intensity (Li and Zachgo, 2013; Viola et al., 2016). In *Lycium ruthenicum*, *LrTCP4* performed as a positive regulator in kukoamine biosynthesis and other secondary metabolites (Chahel et al., 2019). In *Artemisia annua* (*A. annua*), *AaTCP14* and *AaTCP15* were essential for jasmonate (JA)-induced artemisinin biosynthesis (Ma et al., 2018, 2021).

To date, genome-wide identification of the TCP family members has been identified and characterized in many dicots and monocots plants, such as *A. thaliana* (Li, 2015), *Solanum lycopersicum* (Parapunova et al., 2014), *Malus domestica* (Xu et al., 2014), *Citrullus lanatus* (Shi et al., 2016), *Nicotiana tabacum* (Chen et al., 2016), *Fragaria vesca* (Wei et al., 2016), *Glycine max* (Feng et al., 2018), *Gossypium barbadense* (Zheng et al., 2018), *Vitis vinifera* (Leng et al., 2019), *Solanum tuberosum* (Bao et al., 2019), *Phyllostachys edulis* (Liu et al., 2018), *Z. mays* (Ding et al., 2019), *Panicum virgatum* (Zheng et al., 2019), *Hordeum vulgare* subsp. vulgare (Gao et al., 2021), and so on. However, there is no research about the identification and functional characterization of TCP proteins in CPT-producing plants. Here, we first present a detailed and comprehensive analysis of the *TCP* gene family through the whole *O. pumila* genome, such as identification of all TCP family members, phylogenetic relationships, conserved motifs, gene structure, *cis*-elements in gene promoter regions, the expression levels of *O. pumila* TCP (*OpTCP*) genes in diverse tissues, co-expression analysis of key enzymes involved in CPT biosynthesis, and subcellular localization, which could provide valuable information for understanding its classification and functions in *O. pumila*.

## Materials and Methods

### Identification and Characteristics of *Teosinte Branched 1/Cycloidea/Proliferating Cell Factors 1/2* Gene Family in *Ophiorrhiza pumila*

All annotated protein sequences from *O. pumila* genome database^1^ were obtained to comprehensively identify genomic TCP TFs (Rai et al., 2021). Moreover, to avoid missing any *OpTCP* genes, the Hidden Markov Model (HMM) of the TCP domain (Pfam, PF03634) was applied as a query to blast all TCP-containing sequences against our protein database by HMMER3.2 software.^2^ Then, all candidate *OpTCPs* were manually further validate using the online programs of CDD,^3^ Simple Modular Architecture Research Tool (SMART),^4^ and Pfam^5^ confirmed the existence of core domains. Finally, the *TCP* gene members were identified in *O. pumila*, after removing incorrect and redundant predicted proteins. The molecular weights (MWs), amino acid lengths, and isoelectric points (pI) of each OpTCP protein were computed by the ExPASy website^6^ (Gasteiger et al., 2005). The subcellular localization of putative OpTCPs protein was predicted by PSORT^7^ and pLoc-mPlant.^8^ In addition, the sequences of the 24 *Arabidopsis* TCP proteins were downloaded from the *Arabidopsis* Information Resource (TAIR).^9^ The sequences of the 23 *O. sativa* and 18 *Coffea canephora* TCP proteins were retrieved from PlantTFDB database.^10^

### Chromosomal Localization and Gene Duplication

The physical locations of *OpTCP* genes on chromosomes were obtained from *O. pumila* genome database and visualized by TBtools software.^11^ Multiple collinear scanning toolkits (MCScanX) and the Basic Local Alignment Search Tool (BLASTP) methods were used to analyze gene duplication events. Tandem repeats were identified based on the criteria defined in the previous report (Cheng et al., 2016), in which two or more genes should be located within a 100 kbp window and displayed at least 70% sequence similarity. The synonymous relationship between *OpTCP* genes and Arabidopsis, rice, and coffee *TCP* genes was visualized by TBtools software.

### Multiple Sequence Alignment and Phylogenetic Analysis

Multi-sequence alignments of all conserved TCP core domains were determined using DNAMAN 6.0 software. The aligned sequences were visualized with WEBLOGO program^12^ for the conserved amino acid residues analysis. To investigate phylogenetic relationships of OpTCPs and assist their classification, the full-length amino acid sequences of 24 AtTCP, 23 *O. sativa* TCP (OsTCP), 18 *C. canephora* TCP (CcTCP), 16 OpTCP, and some functional TCPs were aligned with CLUSTAL software. The protein sequences of those genes involved in the regulation of special secondary metabolism can be found under the following accession numbers: AtTCP3 (At1g53230), AtTCP15 (At1g69690), AaTCP14 (AYF60463.1), AaTCP15 (QKD77227.1), and MdTCP46 (MDP0000319941). The phylogenetic tree was constructed using Neighbor-joining (NJ) method implemented with MEGA 7.0, and its reliability was tested using bootstrapping with 1,000 replicates (Kumar et al., 2016). The display of the phylogenetic tree was performed by Interactive Tree of Life (iTOL).^13^

### Gene Structure and Motif Composition Analysis

The exon-intron structures of *OpTCP* genes were determined by aligning their genomic sequences with corresponding coding sequences (CDS), while diagrams were visualized with online the program Gene Structure Display Server (GSDS v2.0)^14^ (Hu et al., 2015). Additionally, the conserved motifs of OpTCP proteins were investigated using Multiple Expectation-Maximization for Motif Elicitation online program (MEME v5.1.1)^15^ with the following parameters: the number of motifs searched was set as 10; optimum motif width of 6–100 amino acids; and sites of per motif set to ≥2 and ≤600 (Bailey et al., 2009). Subsequently, the TBtools software was used to display and re-edited the gene structure and conserved motif.

### Promoter *cis*-Acting Elements Analysis

To analyze the promoter regions of the *OpTCPs* and key enzyme genes involved in CPT biosynthesis, genomic DNA sequences in the promoter region (−3,000 to −1 bp) were extracted by the TBtools. Identification of candidate CPT biosynthetic pathway genes in *O. pumila* by sequence identifies with characterized genes from the prestrictosidine biosynthetic pathway in *O. pumila* or *C. roseus* (Supplementary Table 1). These CDS sequences were mapped into the *O. pumila* genome to find the corresponding promoter regions. Subsequently, the promoter sequences of each *OpTCP* gene were scanned by Plantpan 3.0^16^ database to predicate and identify *cis*-acting regulatory elements (Chow et al., 2019). Meanwhile, the identification of TBS elements on the promoter of 15 key enzyme genes in the CPT biosynthetic pathway was scanned with conserved TCP-binding sites by blast methods, such as GGNCCCAC, GGNCC, GCCCR, or G(T/C)GGNCCC (Aggarwal et al., 2010).

### Plant Materials, RNA Extraction, and Quantitative Real-Time PCR Analysis

Sterile *O. pumila* seedlings were cultured on solid B5 medium (pH 5.5) under controlled glasshouse conditions at 25°C and 14 h light/10 h dark photoperiod. The roots, stems, and leaves of 2-month-old *O. pumila* seedlings were collected to detect the tissue expression pattern of the *OpTCP* and CPT biosynthetic pathway genes. All of the samples were immediately frozen in liquid nitrogen and then stored at −80°C until used for RNA extraction.

Total RNA was extracted via the RNApure Plant Kit (Tiangen, China). Then cDNA synthesis was performed with PrimeScript^TM^ II First Strand cDNA Synthesis Kit (Tiangen, China). qRT−PCR was performed by StepOnePlus platform (Bio-Rad, Hercules, CA, United States) with a SYBR Green PCR Master Mix Kit (SYBR^®^ Premix Ex Taq^TM^, Japan). Transcript abundance was calculated relative to *OpUBQ* (Ubiquitin) by 2^–ΔΔCt^ method. Primers were listed in Supplementary Table 2. Co-expression analysis of candidate genes was performed by Pearson’s correlation test, and coefficients >0.8 indicated co-expression. Co-expression of candidate genes has been re-visualized as a network figure by Cytoscape_v3.7.2.

### Dual-Luciferase Assay

To investigate the ability of OpTCP15 to regulate the expression of CPT biosynthesis pathway genes, the full-length coding sequence of *OpTCP15* was amplified and inserted into the pHB-yellow fluorescent protein (YFP) vector (effectors). The promoter regions of *Op7DLH* and *Op8HGO* were ligated into the pGreenII0800-LUC vector (reporters). The Renilla luciferase gene driven by the CaMV 35 S promoter was used as an internal control. Empty pHB-YFP was used as the negative control for the effector. Infiltration and detection were performed as described previously (Hao et al., 2021). The ratio of firefly luciferase to Renilla luciferase represents the relative activity of the promoter. All experiments were repeated three times for each combination. All primers used for these constructs are listed in Supplementary Table 2.

### Subcellular Localization Assay

To investigate the subcellular localization of candidate OpTCP proteins, the full-length coding sequences were inserted into the pHB-YFP vector. The pHB-YFP (empty vector) was used as the negative control. The plasmids pHB-OpTCPs-YFP and pHB-YFP were transformed into the *Agrobacterium tumefaciens* strain GV3101, respectively. Then strains GV3101 harboring OpTCPs-YFP and pHB-YFP were transiently infected the epidermal cells of 5-week-old *N. benthamiana* leaves. YFP fluorescences were analyzed 2 days after infiltration with an LSM880 confocal laser microscope (Carl Zeiss, Germany). Nuclei were stained with 4’ 6-diamidino-2-phenylindole (DAPI, Sigma), and three biological replicates were performed to verify the results (Hao et al., 2021).

## Results

### Identification of Teosinte Branched 1/Cycloidea/Proliferating Cell Factors 1/2 Family Members in the *Ophiorrhiza pumila* Genome

To identify *TCP* genes in *O. pumila*, an HMM search was conducted using the HMM profiles of TCP domain (PF03634) as queries against the *O. pumila* genome dataset (see text footnote 1). A total of 16 non-redundant *TCP* genes were obtained and named as OpTCP1 to OpTCP16 according to their order in the *O. pumila* genomic sequence (Table 1). Each candidate gene was further analyzed to confirm the integrity of the TCP domain of TCP proteins with the online program of CDD, SMART, and Pfam. Meanwhile, MWs, amino acid lengths, pI, and subcellular location of OpTCP proteins were analyzed (Table 1). The MWs of OpTCP proteins ranged from 18.91 (OpTCP1) to 54.25 (OpTCP13) kDa, with an average of 37.51 kDa. The protein lengths were distributed from 174 (OpTCP1) to 504 (OpTCP13) amino acids, and pI varied from 5.60 (OpTCP11) to 10.01 (OpTCP6). They were all predicted to be located in the nucleus.

**TABLE 1:** Detailed information for 16 *OpTCP* genes in the *O. pumila* genome.

ID	Gene name	Type	Chr	Start	Stop	Strand	No. of Exon	CDS (bp)	Protein (aa)	MWs (Da)	pI	Loc
Opuchr01_g0001120-1.1	*OpTCP1*	PCF	chr01	578509	577985	−	1	525	174	18907.37	9.15	Nucleus
Opuchr04_g0008760-1.1	*OpTCP2*	CYC/TB1	chr04	4881540	4880265	−	2	1,179	392	44582.07	8.36	Nucleus
Opuchr04_g0064620-1.1	*OpTCP3*	CIN	chr04	32348228	32347020	−	1	1,209	402	44514.21	7.39	Nucleus
Opuchr05_g0002240-1.1	*OpTCP4*	CIN	chr05	1225789	1226784	+	1	996	331	36943.08	6.37	Nucleus
Opuchr05_g0060100-1.1	*OpTCP5*	CIN	chr05	27883069	27884421	+	1	1,353	450	48977.51	6.59	Nucleus
Opuchr06_g0114980-1.1	*OpTCP6*	PCF	chr06	56622961	56623614	+	1	654	217	23276.48	10.01	Nucleus
Opuchr07_g0000090-1.1	*OpTCP7*	PCF	chr07	67153	67995	+	1	843	280	29639.13	8.69	Nucleus
Opuchr07_g0005290-1.1	*OpTCP8*	CIN	chr07	2963107	2964165	+	1	1,059	352	38817.75	6.79	Nucleus
Opuchr07_g0006620-1.1	*OpTCP9*	CYC/TB1	chr07	3648191	3647069	−	2	1,203	400	44972.86	9.22	Nucleus
Opuchr07_g0038750-1.1	*OpTCP10*	PCF	chr07	19064610	19063813	−	1	798	265	27475.75	9.51	Nucleus
Opuchr09_g0004960-1.1	*OpTCP11*	PCF	chr09	3099165	3100307	+	1	1,143	380	39829.61	5.6	Nucleus
Opuchr09_g0016060-1.1	*OpTCP12*	CIN	chr09	10055733	10054717	−	1	1,017	338	37364.12	6.26	Nucleus
Opuchr09_g0061970-1.1	*OpTCP13*	CIN	chr09	28382461	28380947	−	1	1,515	504	54252.12	7.8	Nucleus
Opuchr10_g0056950-1.1	*OpTCP14*	PCF	chr10	28389738	28390955	+	1	1,218	405	42082.31	7.02	Nucleus
Opuchr11_g0082210-1.1	*OpTCP15*	PCF	chr11	36501516	36502670	+	1	1,155	384	41199.34	7.86	Nucleus
Opuchr11_g0088190-1.1	*OpTCP16*	PCF	chr11	39951958	39952734	+	1	777	258	27359.43	8.57	Nucleus

*AA, amino acid residues; Chr, chromosome; MW, molecular weight; pI, theoretical isoelectric point; Loc, subcellular location; OpTCP, O. pumila TCP.*

### Chromosome Localization and Duplication of the *OpTCP* Gene Family

**FIGURE 1:**
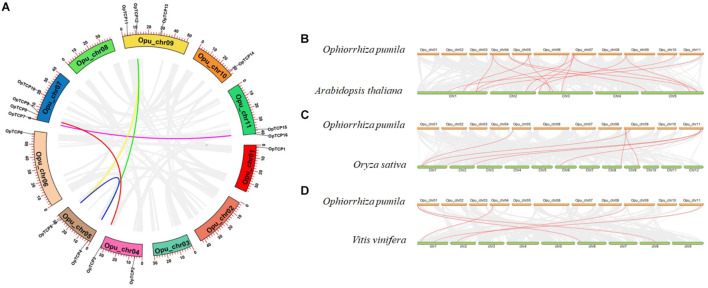
The chromosomal location and synteny analysis of *OpTCP* genes. **(A)** Circos diagram illustrates the chromosomal locations of *OpTCP* genes and their synteny. The colored lines display similarity of different genes. **(B–D)** Synteny analysis of *TCP* genes between *O. pumila* and *Arabidopsis*, and rice, and *V. vinifera*, respectively. TCP, teosinte branched 1/cycloidea/proliferating cell factors 1/2; OpTCP, *O. pumila* TCP.

Sixteen *OpTCP* genes were disproportionately distributed on 8 of 11 *O. pumila* chromosomes (Figure 1A and Supplementary Figure 2). There was no distribution on Chr 2, 3, and 8. Four *OpTCP* genes on Chr 7; three *OpTCP* genes on Chr 9; two on Chr 4, Chr 5, and Chr 11; only one on Chr 1, Chr 6, and Chr 10. The possible relationships with the *OpTCP* genes and potential gene duplication type, collinear analyses were investigated in *O. pumila* genome. As illustrated in Figure 1A, seven genes involved in three segmental duplication events, such as Opu_chr05 (OpTCP4, 5)/Opu_chr09 (OpTCP12), Opu_chr04 (OpTCP3)/Opu_chr07 (OpTCP8), and Opu_chr07 (OpTCP7)/Opu_chr11 (OpTCP16). In contrast, no tandem duplication events were observed, suggesting that segmental duplications were the main causes for the amplification of the *OpTCP* gene family.

### Microsynteny and Evolutionary Patterns of the *OpTCP* Genes in *O. pumila*

To further explore the functions and evolutionary relationships of *TCP* genes, large-scale comparative synteny maps between *O. pumila* and *Arabidopsis*, and *O. sativa* or *V. vinifera* were analyzed at genome-wide levels (Figures 1B–D). As a result, a total of 15 pairs of syntenic *TCP* genes were identified between *O. pumila* and *Arabidopsis* (Figure 1B and Supplementary Table 3), while 7 and 5 pairs of *TCP* genes were identified between *O. pumila*, *O. sativa*, and *V. vinifera* (Figures 1C,D and Supplementary Table 3), respectively. Among the synteny events, *O. pumila* between *Arabidopsis* and 5 *OpTCP* genes were found to be associated with two synteny events, such as OpTCP2-AtTCP12/AtTCP18, OpTCP3-AtTCP5/AtTCP17, and OpTCP5-AtTCP3/AtTCP4 (Supplementary Table 3). Interestingly, four of these five genes were in CIN and CYC/TB1subclade, indicating higher conservation of CIN and CYC/TB1 than PIF subclade in the *TCP* gene family. In addition, the *OpTCP15* gene had a homologous relationship in all three plants, indicating that this gene may have an important role in evolution.

### Phylogenetic Analysis and Classification of *OpTCP* Genes

**FIGURE 2:**
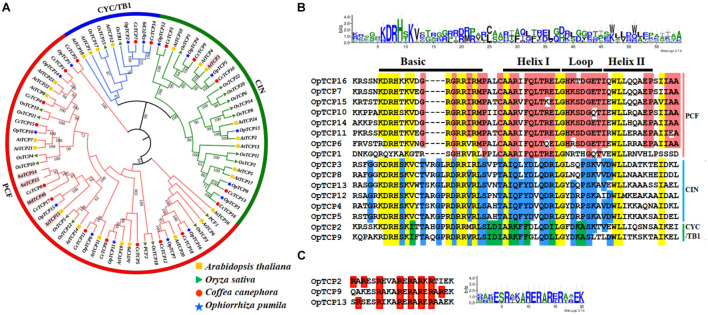
Identification of TCP transcription factor genes in *O. pumila*. **(A)** Phylogenetic analysis of TCP proteins using the un-rooted Neighbor-Joining (NJ) method with 1,000 bootstrap replicates. Five *TCP* genes involved in special secondary metabolism are marked with pink shades. **(B)** The conserved TCP domains analysis using WebLogo and multiple sequence alignments by ClustalW. Each letter represents one amino acid, and the left column corresponds to the name of the gene. The yellow region indicates the highly conserved residues of the OpTCP family members, and the red region indicates residues conserved only in the class I subfamily. The blue region indicates residues conserved in the Class II subfamily. Green indicates the residues conserved in the CYC/TB1 class of proteins. The top black bar represents the conserved domain in TCP proteins. **(C)** The R domain in Class II members of the *OpTCP* gene family. TCP, teosinte branched 1/cycloidea/proliferating cell factors 1/2; OpTCP, *O. pumila* TCP.

To explore the phylogenetic and evolutionary relationship of the *TCP* genes in *O. pumila* and group them with the established subfamilies, an unrooted NJ phylogenetic tree was constructed (Figure 2A). Sixteen *OpTCP* genes were clustered into classes I and II, each of which contained eight members. Additionally, class II could be further divided into the CIN and CYC/TB1 subgroups, which contained 6 (OpTCP3, OpTCP4, OpTCP5, OpTCP8, OpTCP12, and OpTCP13) and two TCP members (OpTCP2 and OpTCP9), respectively.

Multiple sequences alignments showed that two subgroups were distinguished by a four-amino-acids deletion at the N-terminal of class I (Figure 2B). Additional, three class II proteins, OpTCP2 and OpTCP9 from class II CYC/TB1, and OpTCP13 from CIN also shared an R domain at the C-terminus of the TCP domain (Figure 2C).

### Gene Structure and Motif Analysis of *OpTCP* Genes

**FIGURE 3:**
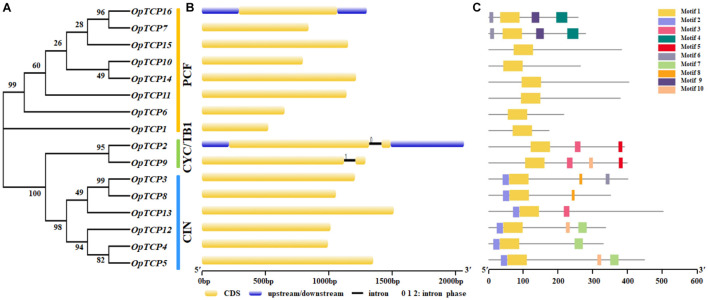
Phylogenetic analysis, gene structure, and conserved motifs of TCP family in *O. pumila*. **(A)** The conserved TCP domain sequences of OpTCP proteins were constructed a Neighbor-Joining phylogenetic tree and the bootstrap test was performed with 1,000 iterations. **(B)** Exon-intron structure of the *OpTCP* genes. Blue indicates untranslated 5′- and 3′-regions, yellow indicates exons; black indicates introns; 0, 1, 2 indicates intron phase. **(C)** Distribution of conserved motifs of OpTCP proteins. Different motifs are shown by different colors numbered 1–10. TCP, teosinte branched 1/cycloidea/proliferating cell factors 1/2; OpTCP, *O. pumila* TCP.

To further understand the pivotal role that exon-intron structural features play in the evolution of *O. pumila* gene families, the structure of *OpTCP* genes was obtained through exon-intron organization analysis. The phylogenetic tree (Figure 3A) revealed the most OpTCP proteins in the same group with similar genetic structures, such as the length and number of the exon. As shown in Figure 3B, class I and class II CIN-type *TCP* genes had only one exon and no intron, while class II CYC/TB1-type *TCP* genes contained two exons. These results suggested that different OpTCP members tended to share different structure organizations.

The conserved motifs of TCP family proteins in *O. pumila* were analyzed by MEME online software, and 10 motifs were identified (Supplementary Table 4). These 10 motifs were distributed across different subgroups in the phylogenetic tree (Figure 3C). For OpTCP proteins, motif 1 was broadly distributed in all OpTCP proteins, which was corresponded to TCP domain. The OpTCPs in subfamily CIN had motif 2 and all members of the CYC/TB1 subfamily and OpTCP13 from CIN contained the motif 3 (R domain). Generally, proteins with similar motif compositions were clustered in the same class indicating that members of the same class may have similar functions.

### Analysis of *cis*-Acting Elements

**FIGURE 4:**
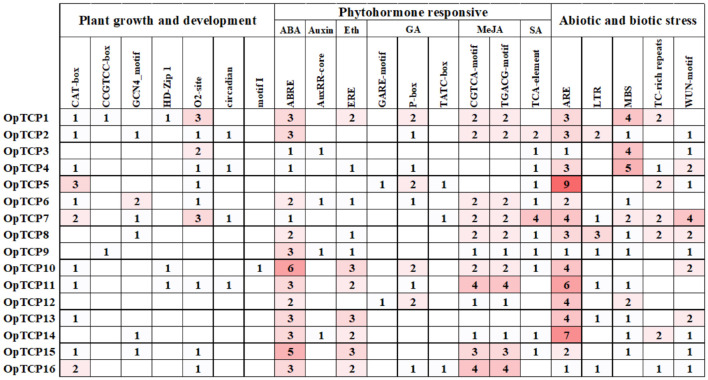
Promoter *cis*-regulatory elements analysis of the *OpTCP* genes. Based on the functional annotation, the *cis*-acting elements were classified into three major classes: plant growth and development, phytohormone responsive, or abiotic and biotic stresses-related *cis*-acting elements (detailed results shown in Supplementary Table 5).

To further investigate the gene function and regulation mechanism of *OpTCP* genes, the *cis*-acting elements in promoter sequences were analyzed by Plantpan 3.0 software. As a result, a variety of *cis*-acting elements involved in plant growth and development, hormone responses, and stress responses were identified. As shown in Figure 4, CAT-box and CCGTCC-box involved in meristem expression were identified in the promoter region of 11 and 2 *OpTCP* genes, respectively. GCN4_motif was related to endosperm expression in plant growth and development. The zein metabolism regulation element (O2-site) and circadian control element (circadian) were found in 10 and 4 *OpTCP* genes, respectively. Additionally, leaf development correlated *cis*-acting regulatory elements (HD-Zip 1) and root-specific (motif I) regulatory elements were also found in the promoter region of the *OpTCP* genes (Supplementary Table 5).

In hormone responses, ABA-responsive *cis*-acting regulatory elements (ABREs) were found in the promoter region of 15 *OpTCP* genes, except for the *OpTCP5* gene. Two MeJA-responsive elements (CGTCA-motif and TGACG-motif) were found in the promoter region of 12 *OpTCP* genes. In addition, auxin-responsive element (AuxRR-core), ethylene-responsive element (ERE), three gibberellin-responsive elements (GARE-motif, P-box, and TATC-box), and salicylic acid-responsive element (TCA-element) were found in the promoter region of 4, 11, 10, and 11 *OpTCP* genes, respectively (Supplementary Table 5).

In stress responses, ARE elements essential for the anaerobic induction were found in the promoter region of 16 *OpTCP* genes. Moreover, low temperature-responsive element (LTR), drought-inducibility element (MBS), TC-rich repeats, and wound-responsive element (WUN-motif) were also found in the promoter region of 7, 13, 7, and 12 *OpTCP* genes, respectively (Supplementary Table 5).

### Expression Patterns of *OpTCP* Genes in Various Tissues

**FIGURE 5:**
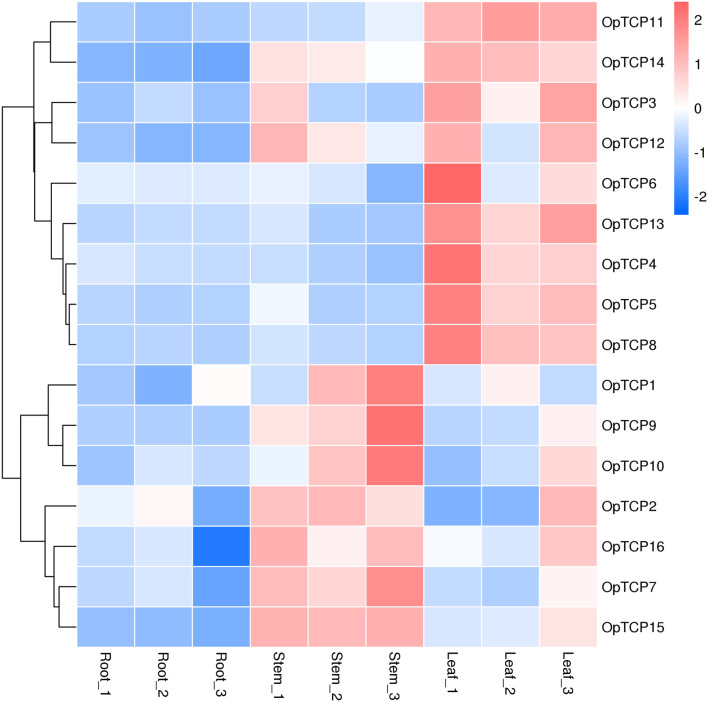
Expression patterns of *OpTCP* genes in different tissues. TCP, teosinte branched 1/cycloidea/proliferating cell factors 1/2; OpTCP, *O. pumila* TCP.

To validate the gene expression profiles, roots, stems, and leaves of *O. pumila* were collected for RNA extraction and quantitative Real-Time PCR (qRT-PCR) analysis. We performed hierarchical clustering with the expression data and accomplished a heatmap to visualize the expression profiles of the *OpTCPs* in different tissues (Figure 5). From the heatmap, most of the *OpTCP* genes preferentially expressed in leaves or stems. For example, 9 *OpTCP* genes (OpTCP3, 4, 5, 6, 8, 11, 12, 13, and 14) had the highest expression levels in leaves, while the other *OpTCPs* (OpTCP1, 2, 7, 9, 10, 15, and 16) showed a higher expression level in stems. In addition, OpTCP7, 10, 14, 15, and 16 consistently had high expression level in all tissues, while OpTCP1, 2, 3, 6, and 12 with low expression level.

### Co-expression Analyses of Candidate Camptothecin Biosynthesis OpTCPs

**FIGURE 6:**
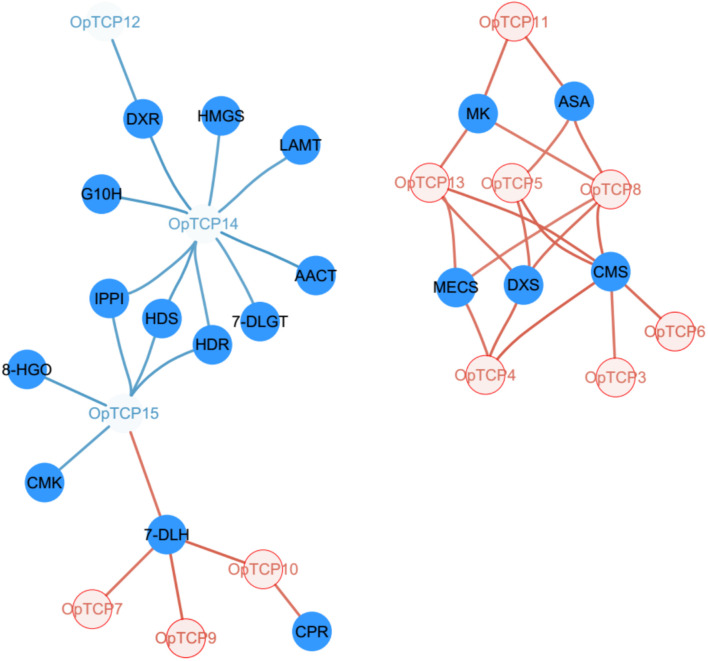
Regulatory network of TCP transcription factors and key CPT-biosynthetic genes. The colored fonts represent TCP TFs and the solid blue circles represent CPT-biosynthetic genes. The edges are drawn when the absolute value of the linear correlation coefficient is >0.8. The blue and red line represents the negative correlation and positive correlation, respectively. TCP, teosinte branched 1/cycloidea/proliferating cell factors 1/2; CPT, camptothecin.

Analysis was conducted to determine the co-expression of all *OpTCPs* with target genes in CPT biosynthesis pathways. The expression levels of key enzyme genes, such as *OpG10H*, *Op10HGO*, *Op8HGO*, *OpSLS1*, *Op7-DLGT*, *OpTDC*, *OpLAMT*, *OpIS*, *OpIO*, and *OpSTR*, were extremely higher in roots while *Op7-DLH* and *OpCPR* showed higher expression levels in stems (Supplementary Figure 3). The gene co-expression analysis revealed that six *OpTCP* (*OpTCP3*, *4*, *5*, *6*, *8*, and *13*) genes were in strong positive correlations with 2-C-methyl-Derythritol 4-phosphate (MEP) pathway genes (Pearson correlation coefficient *r* > 0.8 and *p* < 0.05, Figure 6 and Supplementary Table 6), while *OpTCP12*, *OpTCP14*, and *OpTCP15* genes were in negative correlations with MEP pathway genes. In addition, *OpTCP14* and *OpTCP15* were in strong negative correlations with *OpG10H*, *Op7-DLGT, OpLAMT*, and *Op8HGO* (*r* < −0.8), respectively. Moreover, the Pearson coefficients of four OpTCPs (*OpTCP7*, *9*, *10*, *and 15*) and *Op7-DLH* were >0.8 (Supplementary Table 6). Overall, this result suggested that 12 of 16 *OpTCPs* might be associated with CPT and its precursor biosynthesis.

To identify the OpTCP TFs, which potentially involved in the regulation of CPT biosynthesis, we analyzed the phylogenetic relationships between the candidate OpTCPs and functional TCP TFs that regulated specialized metabolite biosynthesis (i.e., AaTCP14, AaTCP15, AtTCP3, AtTCP15, and MdTCP46). These TFs were grouped into two clades in the neighbor-joining tree (Figure 2A). The OpTCP6, OpTCP7, OpTCP10, OpTCP11, OpTCP14, and OpTCP15 proteins clustered with AaTCP14, AaTCP15, AtTCP15, and MdTCP46 in clade I, whereas AtTCP3 and other OpTCPs belonged to clade II. Interestingly, OpTCP5 and OpTCP15 clustered with AtTCP3 and AtTCP15, respectively, and may be most likely participated in regulating CPT and its precursor biosynthesis.

Finally, to identify the *cis*-elements of TCP TFs, the promoter sequences of the genes encoding enzymes in CPT biosynthesis pathway were analyzed. These results showed that the sequences of GGNCC and GCCCR were identified in the promoter regions of most studied genes (Supplementary Figure 4 and Supplementary Table 7). For example, there were 18, 14, and 9 TBS-binding sites on the promoter sequences of *OpSLS*, *Op8HGO*, and *Op7DLH* genes in CPT biosynthetic pathway, respectively. The finding indicated that the expression of these genes might be regulated by TCP TFs. Overall, *OpTCP5* and *OpTCP15* were likely to have a functional role in CPT and its precursor biosynthesis.

### Dual Luciferase Assay

**FIGURE 7:**
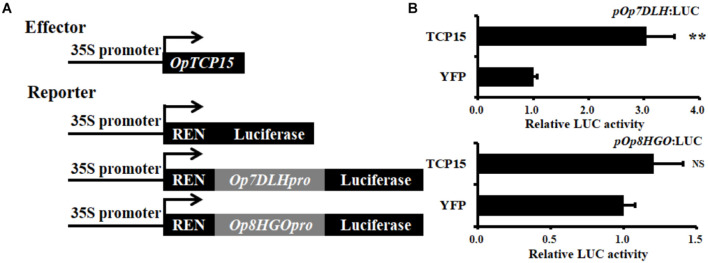
**(A)** Schematic diagrams of the effector and reporter plasmids used in Dual-LUC assay. **(B)** Dual-LUC assay in *N. benthamiana* leaf cells using the constructs shown in panel **(A)**. The relative LUC activity was normalized to that of the reference Renilla (REN) luciferase. Error bars indicate the SD (*n* = 3). Student’s *t*-test: **p < 0.01; NS, no significance; Dual-LUC, dual-luciferase assay.

Subsequently, the dual luciferase (Dual-LUC) assay was performed to verify whether OpTCP15 protein affected the transcription of *Op7DLH* and *Op8HGO* or not. The results showed that *OpTCP15* significantly activated the *Op7DLH* promoter compared to the YFP control, while the *Op8HGO* was slightly upregulated (Figure 7). Together, *OpTCP15* was likely to have a functional role in CPT and its precursor biosynthesis.

### Subcellular Localization Analysis of Selected OpTCPs

**FIGURE 8:**
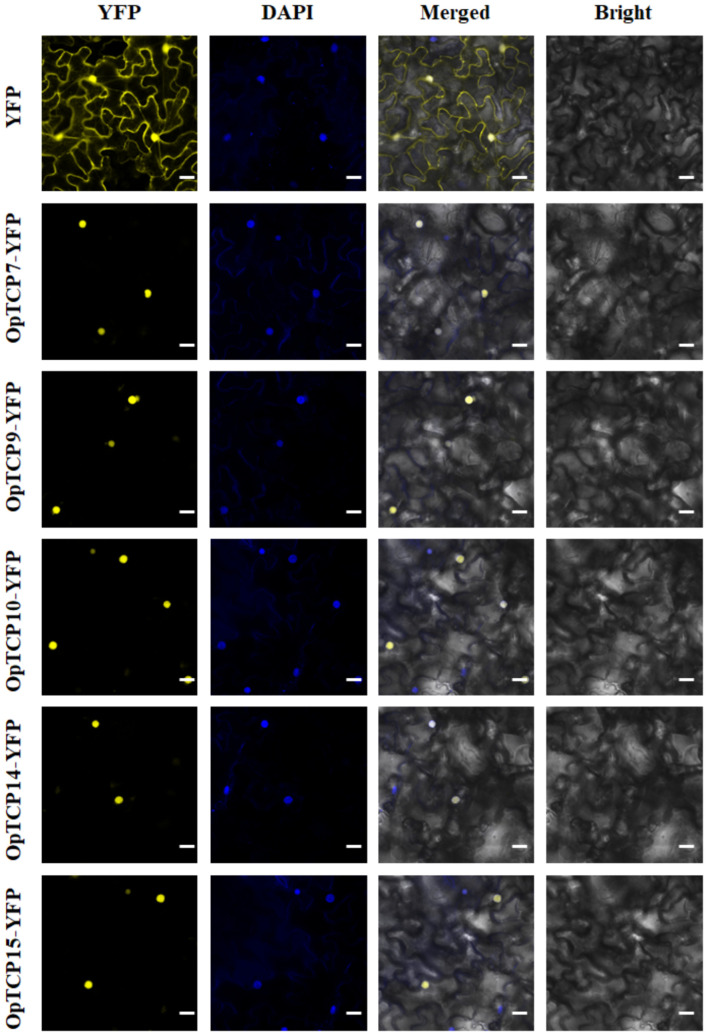
The subcellular localization of the selected five OpTCPs. Scale bars: 20 μm. OpTCP, *O. pumila* TCP.

To determine the subcellular localization of five *OpTCP* genes, which were strongly related to the key enzyme genes of the iridoid pathway, pHB-OpTCP7/9/10/14/15-YFP and pHB-YFP were transiently expressed in *N. benthamiana* leaves. As shown in Figure 8, the *N. benthamiana* leaves transformed with pHB-YFP vector displayed fluorescence in nucleus and cytoplasm. In contrast, all fluorescence in cells transformed with pHB-OpTCP7/9/10/14/15-YFP was detected in the nucleus exclusively, suggesting that all five selected *OpTCP* genes encoded nuclear proteins. It was consistent with their putative role as TFs.

## Discussion

### Evolution and Classification of Teosinte Branched 1/Cycloidea/Proliferating Cell Factors 1/2 Transcription Factor Family

Teosinte branched 1/cycloidea/proliferating cell factors 1/2 genes are a class of terrestrial plant-specific TF, which have been investigated in diverse plants (Liu et al., 2019). However, no systematical study of TCP TFs has been examined in *O. pumila*. In this study, 16 non-redundant *TCP* genes were identified and analyzed from the *O. pumila* genome. Furthermore, a multi-level analysis was performed, such as chromosome location, duplication events, phylogenetic analysis, gene structure, conserved motif, *cis*-acting elements, expression profiles in different tissues, co-expression analysis of key enzymes in CPT biosynthesis, and subcellular localization of candidate TCPs. Compared with those identified *TCP* gene family in the higher plant, the number of *OpTCP* genes was significantly small (Supplementary Table 8). It was consistent with the smaller size of *O. pumila* genome (439.90 Mb) and did not show signs of any recent whole-genome duplication (WGD) in *O. pumila* (Rai et al., 2021). It was found that some *TCP* genes in *O. pumila* had two counterparts in *Arabidopsis* and *O. sativa* (Figures 1B,C), indicating that the deletion of the TCP family in *O. pumila*. Furthermore, compared with the number of *TCP* genes in different subgroups (Supplementary Table 8), our results showed that the PCF subfamily had the largest number of genes, accounting for about 50%, while the CYC/TB1 subfamily had the least number of genes, accounting for about 15%. It was in agreement with the CIN clade being larger than the CYC clade in land plants (Liu et al., 2019).

Phylogenetic analysis and sequence alignment showed that OpTCPs in the same group or subgroup shared similar motifs composition and gene structures (Figure 3), which was consistent with the previously described in *Arabidopsis* (Li, 2015), maize (Ding et al., 2019), potato (Bao et al., 2019), and barley (Gao et al., 2021). For example, motif 2 in N-terminal TCP domain was only present in all CIN subclade, while the conserved R domain (motif 3) was not detected in the PCF subclass (Figure 3C). Additionally, almost all of the *OpTCP* genes within the same subgroup exhibited similar distribution patterns of exon/intron in terms of exon length and intron number. Taken together, the consistency of the motif compositions and the exon/intron structures of *OpTCP* genes further supported the close evolutionary relationships.

### OpTCP Proteins Play Important Roles in Plant Development and Camptothecin Biosynthesis

Increasing evidence indicated that the main function of class I TCP proteins was to promote cell proliferation in leaves (Li et al., 2005; Resentini et al., 2015), while class II proteins played an important role in preventing tissues overgrowth and cell proliferation (Ori et al., 2007). For instance, AtTCP19 and AtTCP20 were class I TCP proteins that are involved in cell division and affect leaf development (Hervé et al., 2009; Danisman et al., 2013). Furthermore, repression of five CIN-like genes (*AtTCP2*, *3*, *4*, *10*, and *24*) in *Arabidopsis* disturbed leaf development (Schommer et al., 2008; Nag et al., 2009). *TB1* mutant increased the number of lateral branches in Maize (Doebley et al., 1995). According to previous studies, syntenic genes between different species may play similar functions (Angiuoli and Salzberg, 2011). In this study, the two class I clade genes *OpTCP7* and *OpTCP11* had orthologous genes *AtTCP20* and *AtTCP19* in *Arabidopsis* (Figure 1B and Supplementary Table 3), respectively, and might be involved in cell division. OpTCP13 was closely homology with AtTCP2 and highly expressed in *O. pumila* leaf (Figure 2A). These findings implied that OpTCP13 may be involved in regulating leaf and flower development. *OpTCP2*, the orthologous gene OsTB1 in *O. sativa*, was highly expressed in *O. pumila* stem, which implied that OpTCP2 played a role in regulating the lateral branching. In addition, qRT-PCR results showed that the expression level of *OpTCPs* in different tissues varied, implying that different *OpTCP* genes may take part in different organ development.

Interestingly, several TCP proteins were found to regulate special metabolism in plants. In *A. annua*, *AaTCP15* promoted artemisinin production by directly binding to and activating the promoters of *ALDH1* and *DBR2*, in which two genes involved in artemisinin biosynthesis (Ma et al., 2021). In this study, co-expression analysis of all *OpTCPs* with target genes in the CPT biosynthesis pathways revealed that nine of the 16 *TCP* genes were in strong correlations with some key enzyme genes in the MEP pathway. Such as the expression patterns of the two genes (*OpDXS* and *OpCMS*) in different tissues are consistent with the *OpTCP5* gene, and the correlation coefficient was >0.8. Additionally, two *TCP* genes (*OpTCP14* and *OpTCP15*) were in strong negative correlations with *OpG10H*, *Op7-DLGT*, *OpLAMT*, and *Op8HGO* (*r* < −0.8), respectively. Four OpTCPs (*OpTCP7*, *9*, *10*, and *15*) and *Op7-DLH* were >0.8 (Supplementary Table 6). Overall, most *OpTCPs* might be involved in CPT the biosynthesis pathway. Additionally, the phylogenetic tree was constructed using the protein sequences of *TCP* family genes from *A. thaliana*, *O. sativa*, *C. canephora*, and *O. pumila*, together with known metabolism-regulating TCPs (AtTCP3, AtTCP15, AaTCP14, AaTCP15, and MdTCP46), to portray their evolutionary relationship. We found that OpTCP15 proteins clustered close with AtTCP15, AaTCP14, AaTCP15, and MdTCP46, while OpTCP5 exhibited a close relationship with AtTCP3 and AtTCP4. Meanwhile, collinearity analyses of *TCP* genes between *O. pumila* and *Arabidopsis* also revealed that *OpTCP5* and *OpTCP15* have a collinear relationship with *AtTCP3*, *AtTCP15*, respectively. In summary, *OpTCP5* and *OpTCP15* were likely to be involved in the biosynthesis process of CPT in *O. pumila*. The functions of two genes are worth exploring in the future.

To identify the *cis*-elements of TCP TFs, the promoter sequences of 15 enzyme-coding genes in CPT biosynthesis were analyzed including three functionally characterized genes (*OpSLS*, *OpTDC*, and *OpSTR*). These results showed that the sequences of GGNCC and GCCCR were identified in the promoter regions of most studied genes (Supplementary Figure 4 and Supplementary Table 7). For example, there were 18, 10, and 7 TBS-binding sites on the promoter sequences of *OpSLS*, *OpSTR*, and *OpTDC* genes, respectively. The finding indicated that TCP TFs may influence the expression of these genes by binding to the TBS binding sites. Furthermore, the results of Dual-LUC assay showed that *OpTCP15* significantly activated the transcription of *Op7DLH* (Figure 7). Investigation on how *OpTCP15* is involved in regulating the biosynthesis of CPT needs to be further conducted. Yeast One-Hybrid (Y1H) and electronic mobility shift assays (EMSAs) were performed to examine OpWRKY2 binding W-box in *O. pumila* (Hao et al., 2021). However, additional methods and techniques are needed to analyze the possible regulatory mechanisms of OpTCP TFs.

## Conclusion

This is the first genome-wide study, such as a systematic analysis of the *OpTCP* gene family in *O. pumila*. A total of 16 TCP family genes were identified and categorized into two classes based on phylogenetics. Expression patterns of all the 16 *OpTCP* and central enzyme genes in CPT biosynthetic pathway were investigated. Combining the results of co-expression, phylogeny analysis, and Dual-LUC assay revealed that *OpTCP15* potentially participated in the regulation of CPT and its precursor biosynthesis. Additionally, a subcellular localization experiment of five *OpTCP* genes showed that they were all localized in the nucleus. These results provided a foundation for further functional characterization of the candidate *OpTCP* genes with the potential to increase CPT production.

## Data Availability Statement

The original contributions presented in the study are included in the article/Supplementary Material, further inquiries can be directed to the corresponding author/s.

## Author Contributions

CW analyzed the data. CW and YW wrote the original draft of this manuscript. XH, MS, GK, and Z-GZ revised the manuscript. All authors have read and approved the final version.

## Conflict of Interest

The authors declare that the research was conducted in the absence of any commercial or financial relationships that could be construed as a potential conflict of interest. The handling editor declared a past collaboration with the author GK.

## Publisher’s Note

All claims expressed in this article are solely those of the authors and do not necessarily represent those of their affiliated organizations, or those of the publisher, the editors and the reviewers. Any product that may be evaluated in this article, or claim that may be made by its manufacturer, is not guaranteed or endorsed by the publisher.

## Footnotes

1
http://pumila.kazusa.or.jp/

2
http://hmmer.org/

3
https://www.ncbi.nlm.nih.gov/Structure/cdd/wrpsb.cgi

4
http://smart.embl-heidelberg.de/#

5
http://pfam.xfam.org/

6
http://web.expasy.org/protparam/

7
https://psort.hgc.jp/

8
http://www.jci-bioinfo.cn/pLoc-mPlant/

9
http://www.Arabidopsis.org/

10
http://planttfdb.gao-lab.org/

11
https://github.com/CJ-Chen/TBtools

12
http://weblogo.berkeley.edu

13
http://itol.embl.de/

14
http://gsds.cbi.pku.edu.cn/

15
http://meme-suite.org/tools/meme

16
http://plantpan.itps.ncku.edu.tw/

## Abbreviations

CPT: camptothecin
TCP: teosinte branched 1/cycloidea/proliferating cell factors 1/2
TB1: teosinte branched 1
CYC: cycloidea
PCF1/2: proliferating cell factors 1/2
bHLH: basic helix-loop-helix
MEP: 2-Cmethyl-Derythritol 4-phosphate.

## References

[B1] Aggarwal P. Gupta M. D. Joseph A. P. Chatterjee N. Srinivasan N. Nath U. (2010). Identification of specific DNA binding residues in the TCP family of transcription factors in *Arabidopsis*. *Plant Cell* 22 1174–1189. 10.1105/tpc.109.066647 20363772 PMC2879757

[B2] Angiuoli S. V. Salzberg S. L. (2011). Mugsy: fast multiple alignment of closely related whole genomes. *Bioinformatics* 27 334–342. 10.1093/bioinformatics/btq665 21148543 PMC3031037

[B3] Bailey T. L. Mikael B. Buske F. A. Martin F. Grant C. E. Luca C. (2009). Meme suite: tools for motif discovery and searching. *Nucleic Acids Res.* 37 W202–W208. 10.1093/nar/gkp335 19458158 PMC2703892

[B4] Bao S. Zhang Z. Lian Q. Sun Q. Zhang R. (2019). Evolution and expression of genes encoding TCP transcription factors in *Solanum tuberosum* reveal the involvement of *StTCP23* in plant defence. *BMC Genet.* 20:91. 10.1186/s12863-019-0793-1 31801457 PMC6892148

[B5] Broholm S. K. Tähtiharju S. Laitinen R. A. Albert V. A. Teeri T. H. Elomaa P. (2008). A TCP domain transcription factor controls flower type specification along the radial axis of the Gerbera (Asteraceae) inflorescence. *Proc. Natl. Acad. Sci. U.S.A.* 105 9117–9122. 10.1073/pnas.0801359105 18574149 PMC2449374

[B6] Chahel A. A. Zeng S. Yousaf Z. Liao Y. Yang Z. Wei X. (2019). Plant-specific transcription factor *LrTCP4* enhances secondary metabolite biosynthesis in *Lycium ruthenicum* hairy roots. *Plant Cell Tissue Organ Culture* 136 323–337. 10.1007/s11240-018-1518-2

[B7] Chen L. Chen Y. Q. Ding A. M. Chen H. Xia F. Wang W. F. (2016). Genome-wide analysis of TCP family in tobacco. *Genet. Mol. Res.* 15:gmr.15027728. 10.4238/gmr.15027728 27323069

[B8] Cheng Y. Yao Z. P. Ruan M. Y. Ye Q. J. Wang R. Q. Zhou G. Z. (2016). In silico identification and characterization of the WRKY gene superfamily in pepper (*Capsicum annuum* L.). *Genet. Mol. Res.* 15 1–12. 10.4238/gmr.15038675 27706772

[B9] Chow C. N. Lee T. Y. Hung Y. C. Li G. Z. Tseng K. C. Liu Y. H. (2019). PlantPAN3. 0: a new and updated resource for reconstructing transcriptional regulatory networks from ChIP-seq experiments in plants. *Nucleic Acids Res.* 47 D1155–D1163. 10.1093/nar/gky1081 30395277 PMC6323957

[B10] Cubas P. Lauter N. Doebley J. Coen E. (1999). The TCP domain: a motif found in proteins regulating plant growth and development. *Plant J.* 18 215–222. 10.1046/j.1365-313X.1999.00444.x 10363373

[B11] Danisman S. Van der Wal F. Dhondt S. Waites R. de Folter S. Bimbo A. (2012). *Arabidopsis* class I and class II TCP transcription factors regulate jasmonic acid metabolism and leaf development antagonistically. *Plant Physiol.* 159 1511–1523. 10.1104/pp.112.200303 22718775 PMC3425195

[B12] Danisman S. van Dijk A. D. J. Bimbo A. van der Wal F. Hennig L. de Folter S. (2013). Analysis of functional redundancies within the *Arabidopsis* TCP transcription factor family. *J. Exp. Bot.* 64 5673–5685. 10.1093/jxb/ert337 24129704 PMC3871820

[B13] Ding S. Cai Z. Du H. Wang H. (2019). Genome-wide analysis of *TCP* family genes in *Zea mays* L. identified a role for *ZmTCP42* in drought tolerance. *Int. J. Mol. Sci.* 20:2762. 10.3390/ijms20112762 31195663 PMC6600213

[B14] Doebley J. Stec A. Gustus C. (1995). Teosinte branched1 and the origin of maize: evidence for epistasis and the evolution of dominance. *Genetics* 141 333–346. 8536981 10.1093/genetics/141.1.333 PMC1206731

[B15] Feng Z. J. Xu S. C. Liu N. Zhang G. W. Hu Q. Z. Gong Y. M. (2018). Soybean TCP transcription factors: evolution, classification, protein interaction and stress and hormone responsiveness. *Plant Physiol. Biochem.* 127 129–142. 10.1016/j.plaphy.2018.03.020 29579640

[B16] Gao G. Kan J. Jiang C. Ahmar S. Zhang J. Yang P. (2021). Genome-wide diversity analysis of TCP transcription factors revealed cases of selection from wild to cultivated barley. *Funct. Integrative Genomics.* 21 31–42. 10.1007/s10142-020-00759-4 33169329

[B17] Gasteiger E. Hoogland C. Gattiker A. Wilkins M. R. Appel R. D. Bairoch A. (2005). *Protein Identification and Analysis Tools on the ExPASy Server. The Proteomics Protocols Handbook.* Berlin: Springer, 571–607.

[B18] Hao X. Xie C. Ruan Q. Zhang X. Wu C. Han B. (2021). The transcription factor *OpWRKY2* positively regulates the biosynthesis of the anticancer drug camptothecin in *Ophiorrhiza pumila*. *Horticulture Res.* 8 1–14. 10.1038/s41438-020-00437-3 33384421 PMC7775441

[B19] Hervé C. Dabos P. Bardet C. Jauneau A. Auriac M. C. Ramboer A. (2009). In vivo interference with *AtTCP20* function induces severe plant growth alterations and deregulates the expression of many genes important for development. *Plant Physiol.* 149 1462–1477. 10.1104/pp.108.126136 19091878 PMC2649380

[B20] Horn S. Pabon-Mora N. Theuß V. S. Busch A. Zachgo S. (2015). Analysis of the CYC/TB1 class of *TCP* transcription factors in basal angiosperms and magnoliids. *Plant J.* 81 559–571. 10.1111/tpj.12750 25557238

[B21] Howarth D. G. Donoghue M. J. (2006). Phylogenetic analysis of the “ECE”(CYC/TB1) clade reveals duplications predating the core eudicots. *Proc. Natl. Acad. Sci. U.S.A.* 103 9101–9106. 10.1073/pnas.0602827103 16754863 PMC1482573

[B22] Hu B. Jin J. Guo A. Y. Zhang H. Luo J. Gao G. (2015). GSDS 2.0: an upgraded gene feature visualization server. *Bioinformatics* 31 1296–1297. 10.1093/bioinformatics/btu817 25504850 PMC4393523

[B23] Johnson A. J. Rajan R. Baby S. (2018). Secondary metabolites from Ophiorrhiza. *Nat. Products J.* 8 248–267. 10.2174/2210315508666180515104735

[B24] Kai G. Wu C. Gen L. Zhang L. Cui L. Ni X. (2015). Biosynthesis and biotechnological production of anti-cancer drug Camptothecin. *Phytochem. Rev.* 14 525–539. 10.1007/s11101-015-9405-5

[B25] Kang M. Fu R. Zhang P. Lou S. Yang X. Chen Y. (2021). A chromosome-level *Camptotheca acuminata* genome assembly provides insights into the evolutionary origin of camptothecin biosynthesis. *Nat. Commun.* 12 1–12. 10.1038/s41467-021-23872-9 34112794 PMC8192753

[B26] Kellner F. Kim J. Clavijo B. J. Hamilton J. P. Childs K. L. Vaillancourt B. (2015). Genome-guided investigation of plant natural product biosynthesis. *Plant J.* 82 680–692. 10.1111/tpj.12827 25759247

[B27] Kosugi S. Ohashi Y. (1997). PCF1 and PCF2 specifically bind to *cis* elements in the rice proliferating cell nuclear antigen gene. *Plant Cell* 9 1607–1619. 10.1105/tpc.9.9.1607 9338963 PMC157037

[B28] Kumar S. Stecher G. Tamura K. (2016). MEGA7: molecular evolutionary genetics analysis version 7.0 for bigger datasets. *Mol. Biol. Evol.* 33 1870–1874. 10.1093/molbev/msw054 27004904 PMC8210823

[B29] Leng X. Wei H. Xu X. Ghuge S. A. Jia D. Liu G. (2019). Genome-wide identification and transcript analysis of *TCP* transcription factors in grapevine. *BMC Genomics* 20:786. 10.1186/s12864-019-6159-2 31664916 PMC6819353

[B30] Li C. Potuschak T. Colón-Carmona A. Gutiérrez R. A. Doerner P. (2005). *Arabidopsis TCP20* links regulation of growth and cell division control pathways. *Proc. Natl. Acad. Sci. U.S.A.* 102 12978–12983. 10.1073/pnas.0504039102 16123132 PMC1200278

[B31] Li S. (2015). The *Arabidopsis thaliana* TCP transcription factors: a broadening horizon beyond development. *Plant Signal. Behav.* 10:e1044192. 10.1080/15592324.2015.1044192 26039357 PMC4622585

[B32] Li S. Zachgo S. (2013). TCP3 interacts with R2R3-MYB proteins, promotes flavonoid biosynthesis and negatively regulates the auxin response in *Arabidopsis thaliana*. *Plant J.* 76 901–913. 10.1111/tpj.12348 24118612

[B33] Liu H. L. Wu M. Li F. Gao Y. M. Chen F. Xiang Y. (2018). TCP transcription factors in moso bamboo (*Phyllostachys edulis*): genome-wide identification and expression analysis. *Front. Plant Sci.* 9:1263. 10.3389/fpls.2018.01263 30344527 PMC6182085

[B34] Liu M. M. Wang M. M. Yang J. Wen J. Guo P. C. Wu Y. W. (2019). Evolutionary and comparative expression analyses of TCP transcription factor gene family in land plants. *Int. J. Mol. Sci.* 20:3591. 10.3390/ijms20143591 31340456 PMC6679135

[B35] Liu Y. Q. Li W. Q. Morris-Natschke S. L. Qian K. Yang L. Zhu G. X. (2015). Perspectives on biologically active camptothecin derivatives. *Med. Res. Rev.* 35 753–789. 10.1002/med.21342 25808858 PMC4465867

[B36] Luo D. Carpenter R. Vincent C. Copsey L. Coen E. (1996). Origin of floral asymmetry in Antirrhinum. *Nature* 383 794–799. 10.1038/383794a0 8893002

[B37] Lupas A. Van Dyke M. Stock J. (1991). Predicting coiled coils from protein sequences. *Science* 252 1162–1164. 10.1126/science.252.5009.1162 2031185

[B38] Ma Y. N. Xu D. B. Li L. Zhang F. Fu X. Q. Shen Q. (2018). Jasmonate promotes artemisinin biosynthesis by activating the TCP14-ORA complex in *Artemisia annua*. *Sci. Adv.* 4:eaas9357. 10.1126/sciadv.aas9357 30627665 PMC6317983

[B39] Ma Y. N. Xu D. B. Yan X. Wu Z. K. Kayani S. I. Shen Q. (2021). Jasmonate-and abscisic acid-activated AaGSW1-AaTCP15/AaORA transcriptional cascade promotes artemisinin biosynthesis in *Artemisia annua*. *Plant Biotechnol. J.* 19 1412–1428. 10.1111/pbi.13561 33539631 PMC8313134

[B40] Manassero N. G. U. Viola I. L. Welchen E. Gonzalez D. H. (2013). *TCP* transcription factors: architectures of plant form. *Biomol. Concepts* 4 111–127. 10.1515/bmc-2012-0051 25436570

[B41] Martino E. Della Volpe S. Terribile E. Benetti E. Sakaj M. Centamore A. (2017). The long story of camptothecin: From traditional medicine to drugs. *Bioorganic Med. Chem. Lett.* 27 701–707. 10.1016/j.bmcl.2016.12.085 28073672

[B42] Martín-Trillo M. Cubas P. (2010). *TCP* genes: a family snapshot ten years later. *Trends Plant Sci.* 15 31–39. 10.1016/j.tplants.2009.11.003 19963426

[B43] Nag A. King S. Jack T. (2009). miR319a targeting of TCP4 is critical for petal growth and development in *Arabidopsis*. *Proc. Natl. Acad. Sci. U.S.A.* 106 22534–22539. 10.1073/pnas.0908718106 20007771 PMC2799693

[B44] Ori N. Cohen A. R. Etzioni A. Brand A. Yanai O. Shleizer S. (2007). Regulation of LANCEOLATE by miR319 is required for compound-leaf development in tomato. *Nat. Genet.* 39 787–791. 10.1038/ng2036 17486095

[B45] Palatnik J. F. Allen E. Wu X. Schommer C. Schwab R. Carrington J. C. (2003). Control of leaf morphogenesis by microRNAs. *Nature* 425 257–263. 10.1038/nature01958 12931144

[B46] Parapunova V. Busscher M. Busscher-Lange J. Lammers M. Karlova R. Bovy A. G. (2014). Identification, cloning and characterization of the tomato TCP transcription factor family. *BMC Plant Biol.* 14:157. 10.1186/1471-2229-14-157 24903607 PMC4070083

[B47] Rai A. Hirakawa H. Nakabayashi R. Kikuchi S. Hayashi K. Rai M. (2021). Chromosome-level genome assembly of *Ophiorrhiza pumila* reveals the evolution of camptothecin biosynthesis. *Nat. Commun.* 12 1–19. 10.1038/s41467-020-20508-2 33452249 PMC7810986

[B48] Rather G. A. Sharma A. Pandith S. A. Kaul V. Nandi U. Misra P. (2018). *De novo* transcriptome analyses reveals putative pathway genes involved in biosynthesis and regulation of camptothecin in *Nothapodytes nimmoniana* (Graham) Mabb. *Plant Mol. Biol.* 96 197–215. 10.1007/s11103-017-0690-9 29270891

[B49] Resentini F. Felipo-Benavent A. Colombo L. Blazquez Rodriguez M. A. Alabadí Diego D. Masiero S. (2015). TCP14 and TCP15 mediate the promotion of seed germination by gibberellins in *Arabidopsis thaliana*. *Mol. Plant* 8 482–485. 10.1016/j.molp.2014.11.018 25655823

[B50] Rohani E. R. Chiba M. Kawaharada M. Asano T. Oshima Y. Mitsuda N. (2016). An MYB transcription factor regulating specialized metabolisms in *Ophiorrhiza pumila*. *Plant Biotechnol.* 33 1–9. 10.5511/plantbiotechnology.15.1117a

[B51] Sadre R. Magallanes-Lundback M. Pradhan S. Salim V. Mesberg A. Jones A. D. (2016). Metabolite diversity in alkaloid biosynthesis: a multilane (diastereomer) highway for camptothecin synthesis in *Camptotheca acuminata*. *Plant Cell* 28 1926–1944. 10.1105/tpc.16.00193 27432874 PMC5006703

[B52] Sarvepalli K. Nath U. (2011). Hyper-activation of the TCP4 transcription factor in *Arabidopsis thaliana* accelerates multiple aspects of plant maturation. *Plant J.* 67 595–607. 10.1111/j.1365-313X.2011.04616.x 21518050

[B53] Schommer C. Palatnik J. F. Aggarwal P. Chételat A. Cubas P. Farmer E. E. (2008). Control of jasmonate biosynthesis and senescence by miR319 targets. *PLoS Biol.* 6:e230. 10.1371/journal.pbio.0060230 18816164 PMC2553836

[B54] Shi M. Gong H. Cui L. Wang Q. Wang C. Wang Y. (2020). Targeted metabolic engineering of committed steps improves anti-cancer drug camptothecin production in *Ophiorrhiza pumila* hairy roots. *Industrial Crops Products* 148:112277. 10.1016/j.indcrop.2020.112277

[B55] Shi P. Guy K. M. Wu W. Fang B. Yang J. Zhang M. (2016). Genome-wide identification and expression analysis of the ClTCP transcription factors in *Citrullus lanatus*. *BMC Plant Biol.* 16:85. 10.1186/s12870-016-0765-9 27072931 PMC4830022

[B56] Udomsom N. Rai A. Suzuki H. Okuyama J. Imai R. Mori T. (2016). Function of AP2/ERF transcription factors involved in the regulation of specialized metabolism in *Ophiorrhiza pumila* revealed by transcriptomics and metabolomics. *Front. Plant Sci.* 7:1861. 10.3389/fpls.2016.01861 28018397 PMC5145908

[B57] Viola I. L. Camoirano A. Gonzalez D. H. (2016). Redox-dependent modulation of anthocyanin biosynthesis by the TCP transcription factor TCP15 during exposure to high light intensity conditions in *Arabidopsis*. *Plant Physiol.* 170 74–85. 10.1104/pp.15.01016 26574599 PMC4704573

[B58] Wei W. Hu Y. Cui M. Y. Han Y. T. Gao K. Feng J. Y. (2016). Identification and transcript analysis of the TCP transcription factors in the diploid woodland *strawberry Fragaria* vesca. *Front. Plant Sci.* 7:1937. 10.3389/fpls.2016.01937 28066489 PMC5177655

[B59] Xie X. Lin W. Liu H. Deng J. Chen Y. Liu H. (2016). Ultrasound-responsive nanobubbles contained with peptide–camptothecin conjugates for targeted drug delivery. *Drug Delivery* 23 2756–2764. 10.3109/10717544.2015.1077289 26289216

[B60] Xu R. Sun P. Jia F. Lu L. Li Y. Zhang S. (2014). Genomewide analysis of TCP transcription factor gene family in *Malus domestica*. *J. Genet.* 93 733–746. 10.1007/s12041-014-0446-0 25572232

[B61] Yang M. Wang Q. Liu Y. Hao X. Wang C. Liang Y. (2021). Divergent camptothecin biosynthetic pathway in *Ophiorrhiza pumila*. *BMC Biol.* 19:122. 10.1186/s12915-021-01051-y 34134716 PMC8207662

[B62] You D. Feng Y. Wang C. Sun C. Wang Y. Zhao D. (2021). Cloning, characterization, and enzymatic identification of a new tryptophan decarboxylase from *Ophiorrhiza pumila*. *Biotechnol. Appl. Biochem.* 68 381–389. 10.1002/bab.1935 32353164

[B63] Zheng A. Sun F. Cheng T. Wang Y. Xie K. Zhang C. (2019). Genome-wide identification of members of the *TCP* gene family in switchgrass (*Panicum virgatum* L.) and analysis of their expression. *Gene* 702 89–98. 10.1016/j.gene.2019.03.059 30928363

[B64] Zheng K. Ni Z. Qu Y. Cai Y. Yang Z. Sun G. (2018). Genome-wide identification and expression analyses of *TCP* transcription factor genes in *Gossypium barbadense*. *Sci. Rep.* 8:14526. 10.1038/s41598-018-32626-5 30266918 PMC6162280

