# Supplementary material for: Genome-Wide Identification and Comparative Analysis of the Teosinte Branched 1/Cycloidea/Proliferating Cell Factors 1/2 Transcription Factors Related to Anti-cancer Drug Camptothecin Biosynthesis in Ophiorrhiza pumila
Source: Front Plant Sci. 2021 Oct 7;12:746648. doi: 10.3389/fpls.2021.746648 (PMC8529195; doi:10.3389/fpls.2021.746648)
Supplement: Supplementary file 1 [file Data_Sheet_1.docx]

Supplementary Material

## Supplementary Figures


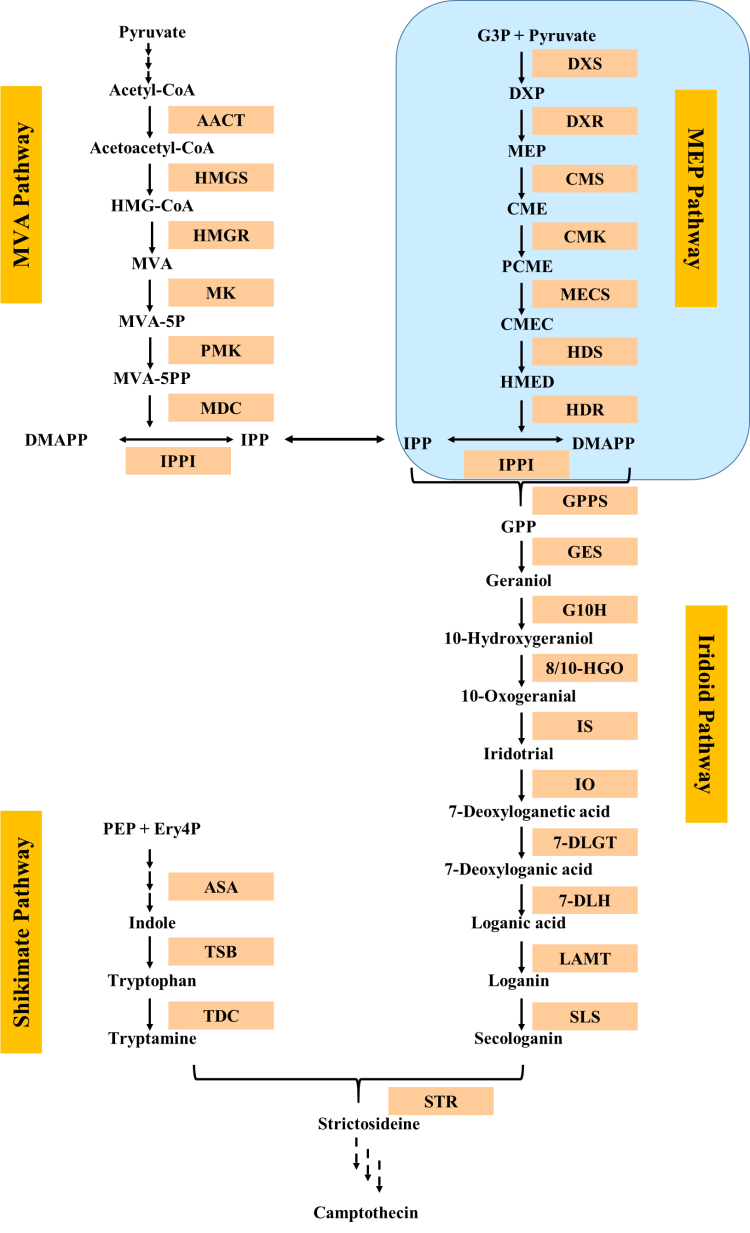


Supplementary Figure 1. A simplified representation of camptothecin biosynthetic pathway.


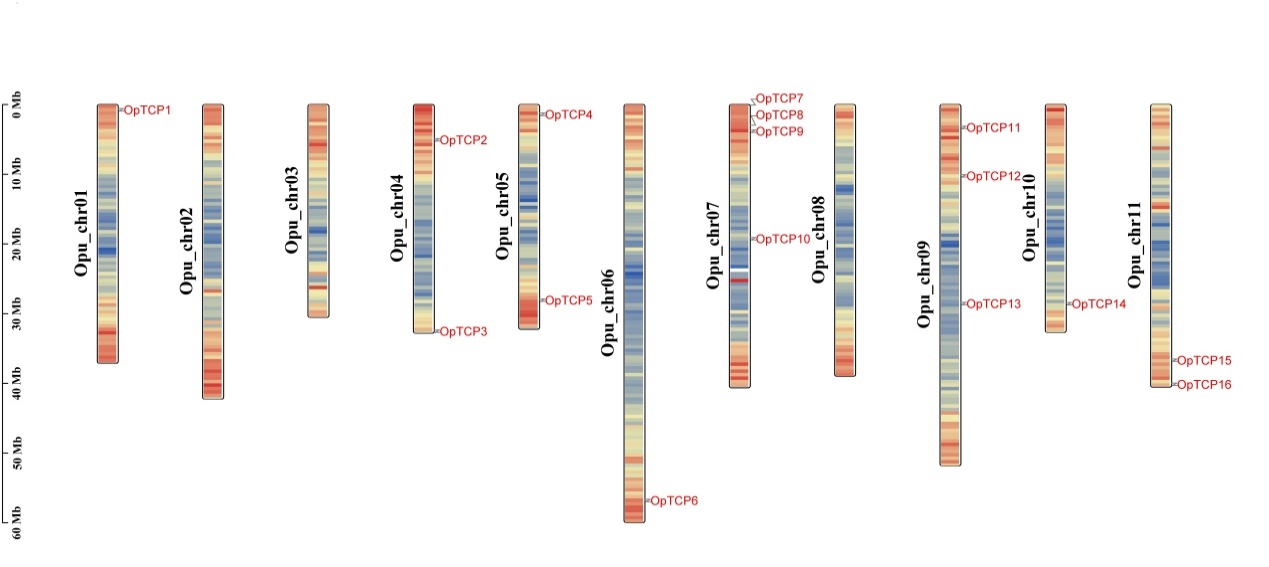


Supplementary Figure 2. Chromosomal location of *OpTCP* genes in *O. pumila*. The chromosomal position of each *OpTCP* was mapped according to the genome of *O. pumila*


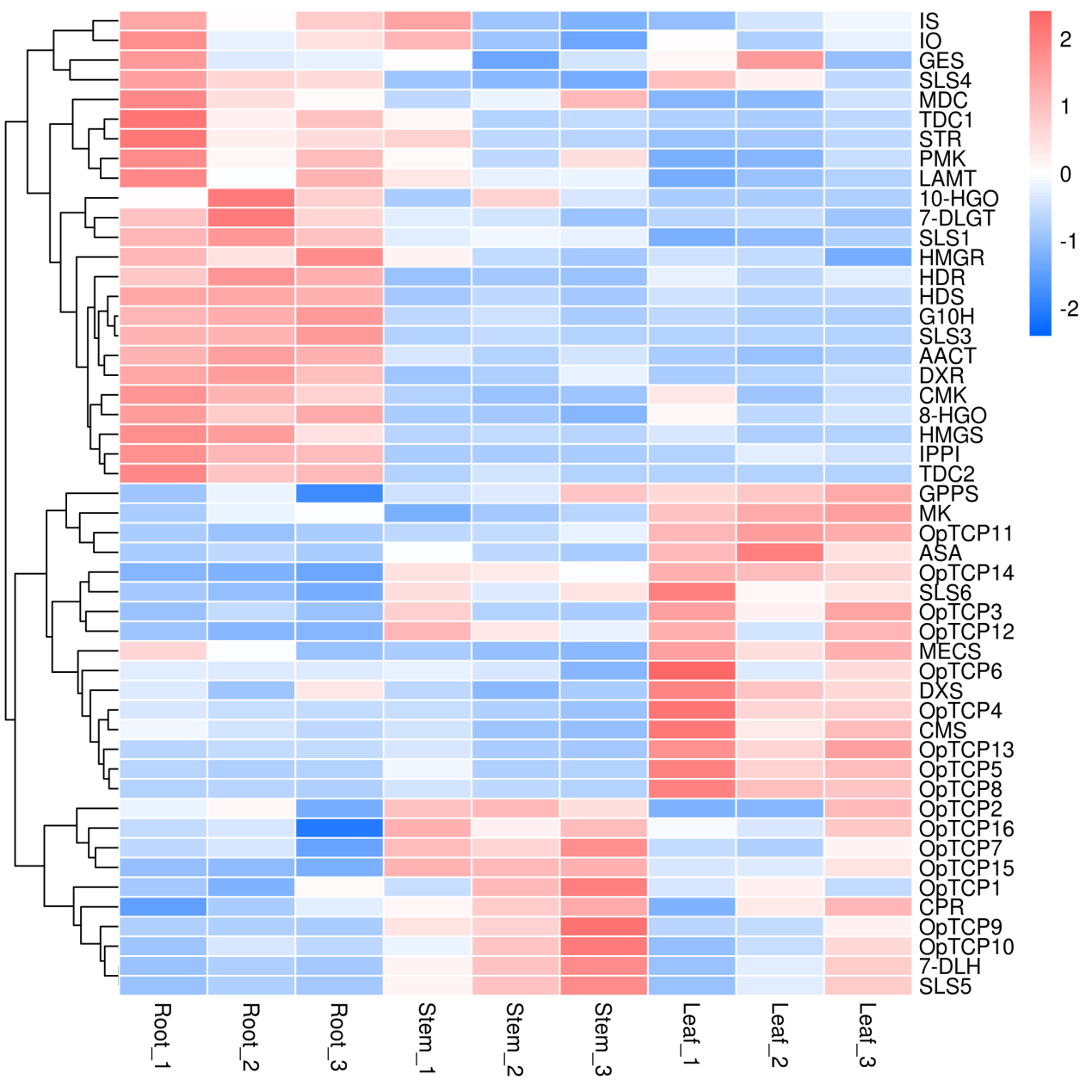


Supplementary Figure 3. Expression patterns of OpTCPs and key CPT-biosynthetic genes in different tissues.

Supplementary Figure 4. TBS elements analysis of key enzyme genes involved in CPT biosynthesis.

## Supplementary Tables

Supplementary table 1. Forty one query protein sequences collected from literatures and GenBank defined as CPT-biosynthese related genes (CPTBGs).

| **Pathway** | **Protein** | **Species** | **Gene Name** | **Enzyme Name** |
| --- | --- | --- | --- | --- |
| **MEP** | ABI35993.1 | *C. roseus* | DXS | 1-deoxy-D-xylulose 5-phosphate synthase 2 |
|  | AAF65154.1 | *C. roseus* | DXR | 1-deoxy-D-xylulose-5-phosphate reductoisomerase |
|  | ACI16377.1 | *C. roseus* | CMS | 4-(cytidine 5-diphospho)-2-C-methylerythritol synthase |
|  | ABI35992.1 | *C. roseus* | CMK | 4-(cytidine 5-diphospho)-2-C-methylerythritolkinase |
|  | AAF65155.1 | *C. roseus* | MECS | 2-C-methylerythritol-2,4-cyclodiphosphate synthase |
|  | AAO24774.1 | *C. roseus* | HDS | hydroxymethylbutenyl 4-diphosphate synthase |
|  | ABI30631.1 | *C. roseus* | HDR | 1-hydroxy-2-methyl-butenyl 4-diphosphate reductase |
|  | ABW98669.1 | *C. roseus* | IPPI | isopentenyl diphosphate isomerase |
| **MVA** | AEC13714.1 | *C. roseus* | AACT | acetyl-CoA : acetyl-CoA C-acetyltransferase |
|  | AEC13715.1 | *C. roseus* | HMGS | 3-hydroxy-3-methylglutaryl-CoA synthase |
|  | AAT52222.1 | *C. roseus* | HMGR | 3-hydroxy-3-methylglutaryl-CoA reductase |
|  | ADR65111.1 | *C. roseus* | MK | mevalonate kinase |
|  | ADR65112.1 | *C. roseus* | PMK | phosphomevalonate kinase |
|  | ADR65113.1 | *C. roseus* | MDC | mevalonate 5-diphosphate decarboxylase |
| **Shikimate** | CAC29060.1 | *C. roseus* | ASA | anthranilate synthase alpha |
|  | CAA01667.1 | *C. roseus* | TDC | tryptophan decarboxylase |
|  | CAA01669.1 | *C. roseus* | TDC | tryptophan decarboxylase |
|  | BAC41515.1 | *O. pumila* | TDC | tryptophan decarboxylase |
|  | QLQ34389.1 | *O. pumila* | TDC2 | tryptophan decarboxylase 2 |
| **Iridoid** | ACC77966.1 | *C. roseus* | GPPS | geranyl diphosphate synthase |
|  | AHA82032.1 | *C. roseus* | GES | geraniol synthase |
|  | BAP90522.1 | *O. pumila* | G10H | geraniol 10-hydroxylase |
|  | CAC80883.1 | *C. roseus* | G10H | geraniol 10-hydroxylase |
|  | AAQ20892.1 | *C. acuminata* | 10-HGO | 10-hydroxygeraniol oxidoreductase |
|  | AHK60836.1 | *C. roseus* | 8-HGO | 8-hydroxy-geraniol oxidoreductase |
|  | AFW98981.1 | *C. roseus* | IS | iridoid synthase |
|  | AHK60833.1 | *C. roseus* | IO | iridoid oxidase (CYP76A26) |
|  | AGX93062.1 | *C. roseus* | 7-DLH | 7-deoxyloganic acid hydroxylase (CYP72A224) |
|  | BAO01109.1 | *C. roseus* | 7-DLGT | 7-deoxyloganetic acid UDP-glucosyltransferase |
|  | AJA37724.1 | *C. roseus* | CPR | NADPH-Cytochrome P450 reductase |
|  | BAC41516.1 | *O. pumila* | CPR | NADPH-Cytochrome P450 reductase |
|  | ABW38009.1 | *C. roseus* | LAMT | loganic acid O-methyltransferase |
|  | QWX38535.1 | *O. pumila* | LAMT | loganic acid methyltransferase |
|  | AAA33106.1 | *C. roseus* | SLS | secologanin synthase |
|  | AGX93064.1 | *C. roseus* | SLS | secologanin synthase |
|  | BAP90521.1 | *O. pumila* | SLS | secologanin synthase |
|  | QWX38538.1 | *O. pumila* | SLS3 | secologanin synthase |
|  | QWX38539.1 | *O. pumila* | SLS4 | secologanin synthase |
|  | QWX38540.1 | *O. pumila* | SLS5 | secologanin synthase |
|  | QWX38541.1 | *O. pumila* | SLS6 | secologanin synthase |
| **DN-CPT** | CAA43936.1 | *C. roseus* | STR | strictosidine synthase |
|  | BAB47180.1 | *O. pumila* | STR | strictosidine synthase |

Supplementary table 2. Primers list used in this study

| **Primer name** | **Squences（5'to3'）** | **Usage** |  |
| --- | --- | --- | --- |
| OpTCP1-qrtF | GCACAACTCGGGACCAACTCAC | qRT-PCR |  |
| OpTCP1-qrtR | TTTCGCCTTGTACTGTCGCTGTC |  |  |
| OpTCP2-qrtF | TCCAGCAGCAACCTTTTACACCTC |  |  |
| OpTCP2-qrtR | TCGGGCATTCGGGCACATATTTC |  |  |
| OpTCP3-qrtF2 | TCAGGCAAAGCAAGAAGGA |  |  |
| OpTCP3-qrtR2 | AAATGAACGCGAAACTCGA |  |  |
| OpTCP4-qrtF | TGGGCTCTCACTTCACACTTTGC |  |  |
| OpTCP4-qrtR | CTCCTTCACCAGCACCATTCCAAG |  |  |
| OpTCP5-qrtF | GGCGAGATCGTGGAAGTTCAAGG |  |  |
| OpTCP5-qrtR | GAATGGCAGTGTGGGCAGAGAG |  |  |
| OpTCP6-qrtF | TCCCGAAGATGGCTGTCACCTC |  |  |
| OpTCP6-qrtR | GCACCCTGGTCATCGCTAAAGC |  |  |
| OpTCP7-qrtF | GCAGAGGGAGGAGGATCAGGATG |  |  |
| OpTCP7-qrtR | GGTGGCGGCAATAATAGAGGGTTC |  |  |
| OpTCP8-qrtF | GCCATTGCCATCAACAGTGTCTTC |  |  |
| OpTCP8-qrtR | ATGGTCTACCACCGGCATACTCC |  |  |
| OpTCP9-qrtF2 | TTTACAGCCCAAGGTCCAAG |  |  |
| OpTCP9-qrtR2 | TCAGCCAATCTAGCGTCAGG |  |  |
| OpTCP10-qrtF | ACGAGGGAGCTGGGTCACAAG |  |  |
| OpTCP10-qrtR | CACGGCAGACACGGTAGAGAAAC |  |  |
| OpTCP11-qrtF2 | CGCCTCCCGAAGAACAGTC |  |  |
| OpTCP11-qrtR2 | CACCAACGGCAGCAACCT |  |  |
| OpTCP12-qrtF | TCTGCACTGCTAGAGGTCCAAGG |  |  |
| OpTCP12-qrtR | GGCTTTGCTGGGACGGTCATAG |  |  |
| OpTCP13-qrtF | GGATGAGCAGTGCCCTGATGAAG |  |  |
| OpTCP13-qrtR | GCCACCCACAAAGCCTACCAAG |  |  |
| OpTCP14-qrtF2 | CTTCATCTTCCGACCCTTCC |  |  |
| OpTCP14-qrtR2 | CGGTTTCTTGGCAACTTTACTG |  |  |
| OpTCP15-qrtF | AAATGGTGGTGGTGGAAGTTCTGG |  |  |
| OpTCP15-qrtR | AGCTCCGCCGCCTGGATTAG |  |  |
| OpTCP16-qrtF | AATTATTGCCGCCACAGGTACAGG |  |  |
| OpTCP16-qrtR | CCCAATTCATCCGAATCCCCATCC |  |  |
| OpUBQ-qrtF | TTTTGCTGGGAAACAGTTGGAAG |  |  |
| OpUBQ-qrtR | CAGAAACCACCACGGAGACGC |  |  |
| OpAACT-qrtF | TGCCCAAACAAGTGGTGCT |  |  |
| OpAACT-qrtR | TCTCGCCACTGACGAGCAC |  |  |
| OpHMGS-qrtF | TGGGTTGGGACAAGATTGCATGG |  |  |
| OpHMGS-qrtR | AGAAGTGAAGTCACGGCTGTCAAG |  |  |
| OpHMGR-qrtF | CAGACCTAATTGGGATGTGGAG |  |  |
| OpHMGR-qrtR | ATTCTTGAGGCGTAATGAGTGGA |  |  |
| OpMK-qrtF | AAATTGACGGGAGCTGGTG |  |  |
| OpMK-qrtR | GGACGAACCGCTGAAAGAA |  |  |
| OpPMK-qrtF | ATGCGGAAGATGGGTAAGGC |  |  |
| OpPMK-qrtR | CCAGGAACACCAGCCAACAAA |  |  |
| OpMDC-qrtF | CGCAGTCTTTATGGTGGATTTGTC |  |  |
| OpMDC-qrtR | TCCTTCTGCCGTGAACTTACC |  |  |
| OpDXS-qrtF | ATGGAAATCTAAAGTGGAGGGC |  |  |
| OpDXS-qrtR | TTCACCGATTAGGGATAACACAGT |  |  |
| OpDXR-qrtF | GCTGGTTCAAATGTGACTCTTCTT |  |  |
| OpDXR-qrtR | CCTCAATGACACCCTGTTCTCC |  |  |
| OpCMS-qrtF | GTTCCGTTGCTTTCATCTACTTC |  |  |
| OpCMS-qrtR | GAGACGCTTCTCTCTTTAACCACT |  |  |
| OpCMK-qrtF | TCCAGGAGTTCCCCTTGATGAC |  |  |
| OpCMK-qrtR | CATTGCTGCTACCACCACCTAA |  |  |
| OpMECS-qrtF | ATTCCGATGGTGATGTGTTGC |  |  |
| OpMECS-qrtR | GGCGTCTAAGTTTCCAAGTTCAT |  |  |
| OpHDS-qrtF | TGGAGGCATACCGACTTCTTG |  |  |
| OpHDS-qrtR | TCTATCTCCTCTTCTGGGGGTTC |  |  |
| OpHDR-qrtF | CGCAAAGGATTCGGACACAA |  |  |
| OpHDR-qrtR | TCAACACCCCAACAGAAACCATA |  |  |
| OpIPPI-qrtF | CCTGCTGTAGCCATCCTCTGTA |  |  |
| OpIPPI-qrtR | ATCAAGTTCATGCTCTCCCCAC |  |  |
| OpGPPS-qrtF | GAGCAGCCCAGTTGTTGAGG |  |  |
| OpGPPS-qrtR | TAAGAGTACCGTTGGCCGAAA |  |  |
| OpGES-qrtF | TATGTCCCTCGTGGTGTTCCT |  |  |
| OpGES-qrtR | CATCGGCAGGCACATACAAAT |  |  |
| OpG10H-qrtF | TGAGGAAGCCGATGTTTCCC |  |  |
| OpG10H-qrtR | TTTCATCACGCCCAATTGCC |  |  |
| Op8HGO-qrtF | AAAGATGTGACTGGAGGATTAGG |  |  |
| Op8HGO-qrtR | TCCCTTCAGAACTCTACCACACA |  |  |
| Op10HGO-qrtF | GGAATTGTTGGTTTGGGTGGAT |  |  |
| Op10HGO-qrtR | TGGCCTCCTCCTTCTTGTGAG |  |  |
| OpIS-qrtF | TAGCCGAAATCCTCCCACTC |  |  |
| OpIS-qrtR | GATTAGCCCATGTCACCCAGA |  |  |
| OpIO-qrtF | AGTTTGGACCTGTAGTCTGGCTTC |  |  |
| OpIO-qrtR | GAAAGGTCGTCAGGGTATCAGG |  |  |
| Op7DLGT-qrtF | GCCATGTCAACTCCATGCTCAGG |  |  |
| Op7DLGT-qrtR | GGTGTGCTTGAGGAGGCGATTATG |  |  |
| Op7DLH-qrtF | GCATTATCAACAATAGAGCGAAGG |  |  |
| Op7DLH-qrtR | TGTCTCCTGTCCAGCCAAGT |  |  |
| OpCPR-qrtF | GGATGAGTACGCACAAGGGATT |  |  |
| OpCPR-qrtR | CTGGGCGAAGATGAGATAGAATAA |  |  |
| OpLAMT-qrtF | TTCACTGGCTTTCCAAGGTTC |  |  |
| OpLAMT1-qrtR | TGAATCACCATCAATCCTCCTC |  |  |
| OpSLS1-qrtF | AAGCATCCCGAATGGCAAGA |  |  |
| OpSLS1-qrtR | GAAGCATCACCGTTGGCATC |  |  |
| OpSLS3-qrtF | ATTGAGGTTGGCGGTGGCTA |  |  |
| OpSLS3-qrtR | GCTTGCGGAAGATGACAGGAG |  |  |
| OpSLS4-qrtF | TGGCATGACCATCCAAGACG |  |  |
| OpSLS4-qrtR | TAGCACGAGACTGCCAATCC |  |  |
| OpSLS5-qrtF | AGGGGGTTTTGAAGGCAACA |  |  |
| OpSLS5-qrtR | AAGCGACGCAAAACTAGTGC |  |  |
| OpSLS6-qrtF | GCAACAACGAGCCGAACTTT |  |  |
| OpSLS6-qrtR | GCAGGCAGAAAGAGTTGCAC |  |  |
| OpASA-qrtF | GAACCATTTCAATTACCACCTCC |  |  |
| OpASA-qrtR | AAACAACGATAAGCCAGCACC |  |  |
| OpTDC1-qrtF | AGCTGCACCATTCAGACCACTTG |  |  |
| OpTDC1-qrtR | AGGCTCAACTTGGCTCAAAACTGG |  |  |
| OpTDC2-qrtF | CCCGAAGAGTTCAGGAAACAAGCC |  |  |
| OpTDC2-qrtR | GGCTCAACTTGGCTGAGAACTGG |  |  |
| OpSTR1-qrtF | TGTCGATTCTTTGTGCCCTTTTC |  |  |
| OpSTR1-qrtR | GCGTATGCGTTTGGTCCGTAG |  |  |
| OpTCP7-F | ATGGGTAGTTCCGAAAACAAGC | Clone |  |
| OpTCP7-R | CTACGATCCTGATCCTTGAGAATC |  |  |
| OpTCP9-F | ATGATCTCTTCCAGTAATGGGATC |  |  |
| OpTCP9-R | TCAATCAATTGACATGCTCTCG |  |  |
| OpTCP10-F | ATGTCATCGGCCTCAGCA |  |  |
| OpTCP10-R | TCAACGGGCATCTCCTTCT |  |  |
| OpTCP14-F | ATGGAATTTATGGAAACACAT |  |  |
| OpTCP14-R | TTATTGAGAATCTTTAGGATTTT |  |  |
| OpTCP15-F | ATGGATGCTGATAATGTTCGTG |  |  |
| OpTCP15-R | CTACGAGTGTCGGTGAGAACTAGT |  |  |
| pHB-OpTCP7-BamHI-F | ctctctctcaagcttggatccATGGGTAGTTCCGAAAACAAGC | Subcellular localization |  |
| pHB-OpTCP7-SpeI-R | gcccttgctcaccatactagtCGATCCTGATCCTTGAGAATC |  |  |
| pHB-OpTCP9-BamHI-F | ctctctctcaagcttggatccATGATCTCTTCCAGTAATGGGATC |  |  |
| pHB-OpTCP9-SpeI-R | gcccttgctcaccatactagtATCAATTGACATGCTCTCG |  |  |
| pHB-OpTCP10-BamHI-F | ctctctctcaagcttggatccATGTCATCGGCCTCAGCA |  |  |
| pHB-OpTCP10-SpeI-R | gcccttgctcaccatactagtACGGGCATCTCCTTCT |  |  |
| pHB-OpTCP14-BamHI-F | ctctctctcaagcttggatccATGGAATTTATGGAAACACAT |  |  |
| pHB-OpTCP14-SpeI-R | gcccttgctcaccatactagtTTGAGAATCTTTAGGATTTT |  |  |
| pHB-OpTCP15-BamHI-F | ctctctctcaagcttggatccATGGATGCTGATAATGTTCGTG |  |  |
| pHB-OpTCP15-SpeI-R | gcccttgctcaccatactagtCGAGTGTCGGTGAGAACTAGT |  |  |
| 0800-pOp7DLH-F | cttgatatcgaattcctgcagAGCAGGTCTTCAACATGGATTACA | Dual-luciferase |  |
| 0800-pOp7DLH-R | cgctctagaactagtggatccTTTAGAAAGAAGGAACTATGGTGAGG |  | |
| 0800-pOp8HGO-F | cttgatatcgaattcctgcagGTAAGCCAAGAAAGTGAAATCAGGA |  |  |
| 0800-pOp8HGO-R | cgctctagaactagtggatccGTTGATCGTCCGGCTAAATGTG |  |  |

Supplementary table 3. Synteny analysis of *TCP* genes between *O. pumila* and *Arabidopsis*, *O. sativa* and *V. vinifera*, respectively.

| **Gene_name** | **Op_Chrom** | **Gene_name** | **Chrom** | **Gene_ID** | **Species** | **Type** |
| --- | --- | --- | --- | --- | --- | --- |
| *OpTCP9* | Opu_chr07 | *AtTCP1* | Chr1 | AT1G67260.1.TAIR10 | *A. thaliana* | CYC/TB1 |
| *OpTCP6* | Opu_chr06 | *AtTCP11* | Chr2 | AT2G37000.1.TAIR10 | *A. thaliana* | PCF |
| *OpTCP2* | Opu_chr04 | *AtTCP12* | Chr1 | AT1G68800.1.TAIR10 | *A. thaliana* | CYC/TB1 |
| *OpTCP8* | Opu_chr07 | *AtTCP13* | Chr3 | AT3G02150.2.TAIR10 | *A. thaliana* | CIN |
| *OpTCP15* | Opu_chr11 | *AtTCP15* | Chr1 | AT1G69690.1.TAIR10 | *A. thaliana* | PCF |
| *OpTCP3* | Opu_chr04 | *AtTCP17* | Chr5 | AT5G08070.1.TAIR10 | *A. thaliana* | CIN |
| *OpTCP2* | Opu_chr04 | *AtTCP18* | Chr3 | AT3G18550.1.TAIR10 | *A. thaliana* | CYC/TB1 |
| *OpTCP11* | Opu_chr09 | *AtTCP19* | Chr5 | AT5G51910.1.TAIR10 | *A. thaliana* | PCF |
| *OpTCP7* | Opu_chr07 | *AtTCP20* | Chr3 | AT3G27010.1.TAIR10 | *A. thaliana* | PCF |
| *OpTCP14* | Opu_chr10 | *AtTCP23* | Chr1 | AT1G35560.1.TAIR10 | *A. thaliana* | PCF |
| *OpTCP5* | Opu_chr05 | *AtTCP3* | Chr1 | AT1G53230.1.TAIR10 | *A. thaliana* | CIN |
| *OpTCP5* | Opu_chr05 | *AtTCP4* | Chr3 | AT3G15030.1.TAIR10 | *A. thaliana* | CIN |
| *OpTCP3* | Opu_chr04 | *AtTCP5* | Chr5 | AT5G60970.1.TAIR10 | *A. thaliana* | CIN |
| *OpTCP8* | Opu_chr07 | *AtTCP5* | Chr5 | AT5G60970.1.TAIR10 | *A. thaliana* | CIN |
| *OpTCP11* | Opu_chr09 | *AtTCP9* | Chr2 | AT2G45680.1.TAIR10 | *A. thaliana* | PCF |
| *OpTCP12* | Opu_chr09 | *OsTCP1* | Chr1 | LOC_Os01g11550.1.MSUv7.0 | *O. sativa* | CIN |
| *OpTCP4* | Opu_chr05 | *OsTCP1* | Chr1 | LOC_Os01g11550.1.MSUv7.0 | *O. sativa* | CIN |
| *OpTCP15* | Opu_chr11 | *OsTCP12* | Chr6 | LOC_Os06g12230.1.MSUv7.0 | *O. sativa* | PCF |
| *OpTCP11* | Opu_chr09 | *OsTCP18* | Chr9 | LOC_Os09g34950.1.MSUv7.0 | *O. sativa* | PCF |
| *OpTCP16* | Opu_chr11 | *OsTCP3* | Chr1 | LOC_Os01g69980.1.MSUv7.0 | *O. sativa* | PCF |
| *OpTCP15* | Opu_chr11 | *OsTCP5* | Chr2 | LOC_Os02g51280.1.MSUv7.0 | *O. sativa* | PCF |
| *OpTCP11* | Opu_chr09 | *PCF2* | Chr8 | LOC_Os08g43160.1.MSUv7.0 | *O. sativa* | PCF |
| *OpTCP1* | Opu_chr01 | - | Chr1 | rna-GSCOC_T00024181001 | *V. vinifera* | PCF |
| *OpTCP15* | Opu_chr11 | - | Chr11 | rna-GSCOC_T00032218001 | *V. vinifera* | PCF |
| *OpTCP16* | Opu_chr11 | - | Chr11 | rna-GSCOC_T00038288001 | *V. vinifera* | PCF |
| *OpTCP6* | Opu_chr06 | - | Chr6 | rna-GSCOC_T00023718001 | *V. vinifera* | PCF |
| *OpTCP8* | Opu_chr07 | - | Chr7 | rna-GSCOC_T00039684001 | *V. vinifera* | CIN |

Supplementary table 4. The details of 10 motifs in the protein sequences of OpTCPs

| **Domain** | **E-value** | **Sites** | **Width** | **Multilevel consensus sequence** |
| --- | --- | --- | --- | --- |
| 1 | 2.1e-390 | 16 | 57 | SKRHTKVGGRGRRVRLPALCAARFFQLTDELGHKSPSKTIEWLLKQAEPAIIALTGT |
| 2 | 1.80E-18 | 6 | 19 | JIEPRGGRISRATGGKDRH |
| 3 | 3.00E-05 | 3 | 17 | ESRAKARERARERAAEK |
| 4 | 1.40E+00 | 2 | 33 | QQHPGLELGLSQEGQIGVLNFQALQQFYQQMGQ |
| 5 | 1.50E+00 | 2 | 12 | QFCGKPWEECNN |
| 6 | 2.10E+00 | 3 | 12 | ENKPAEIKDFQI |
| 7 | 3.10E+00 | 3 | 25 | FGQNQLFSQREPLQSSYSPSIHAWM |
| 8 | 8.10E+01 | 2 | 9 | LPYNPFIRW |
| 9 | 8.70E+01 | 2 | 23 | MGGRMNWPMLGENJGRPHMPTAA |
| 10 | 3.00E+01 | 3 | 12 | EVGRFQKVGPWN |

Supplementary table 5. Cis-acting elements analysis of *OpTCP* genes in this study

| **Gene name** | **Element** | **Start** | **Strand** | **Function** |
| --- | --- | --- | --- | --- |
| *OpTCP1* | CAT-box | 567 | - | cis-acting regulatory element related to meristem expression |
| *OpTCP1* | CCGTCC-box | 455 | - |  |
| *OpTCP1* | HD-Zip 1 | 339 | + | element involved in differentiation of the palisade mesophyll cells |
| *OpTCP1* | O2-site | 266 | - | cis-acting regulatory element involved in zein metabolism regulation |
| *OpTCP1* | O2-site | 310 | - | cis-acting regulatory element involved in zein metabolism regulation |
| *OpTCP1* | O2-site | 1259 | + | cis-acting regulatory element involved in zein metabolism regulation |
| *OpTCP1* | ABRE | 884 | - | cis-acting element involved in the abscisic acid responsiveness |
| *OpTCP1* | ABRE | 1069 | + | cis-acting element involved in the abscisic acid responsiveness |
| *OpTCP1* | ABRE | 1363 | + | cis-acting element involved in the abscisic acid responsiveness |
| *OpTCP1* | CGTCA-motif | 2423 | + | cis-acting regulatory element involved in the MeJA-responsiveness |
| *OpTCP1* | CGTCA-motif | 2469 | + | cis-acting regulatory element involved in the MeJA-responsiveness |
| *OpTCP1* | ERE | 785 | + |  |
| *OpTCP1* | ERE | 2099 | + |  |
| *OpTCP1* | P-box | 464 | - | gibberellin-responsive element |
| *OpTCP1* | P-box | 1459 | + | gibberellin-responsive element |
| *OpTCP1* | TGACG-motif | 2423 | - | cis-acting regulatory element involved in the MeJA-responsiveness |
| *OpTCP1* | TGACG-motif | 2469 | - | cis-acting regulatory element involved in the MeJA-responsiveness |
| *OpTCP1* | ARE | 91 | - | cis-acting regulatory element essential for the anaerobic induction |
| *OpTCP1* | ARE | 100 | - | cis-acting regulatory element essential for the anaerobic induction |
| *OpTCP1* | ARE | 2953 | + | cis-acting regulatory element essential for the anaerobic induction |
| *OpTCP1* | MBS | 87 | - | MYB binding site involved in drought-inducibility |
| *OpTCP1* | MBS | 1043 | - | MYB binding site involved in drought-inducibility |
| *OpTCP1* | MBS | 1704 | + | MYB binding site involved in drought-inducibility |
| *OpTCP1* | MBS | 1980 | + | MYB binding site involved in drought-inducibility |
| *OpTCP1* | TC-rich repeats | 2213 | + | cis-acting element involved in defense and stress responsiveness |
| *OpTCP1* | TC-rich repeats | 2386 | - | cis-acting element involved in defense and stress responsiveness |
| *OpTCP2* | CAT-box | 2162 | - | cis-acting regulatory element related to meristem expression |
| *OpTCP2* | GCN4_motif | 2127 | + | cis-regulatory element involved in endosperm expression |
| *OpTCP2* | O2-site | 843 | - | cis-acting regulatory element involved in zein metabolism regulation |
| *OpTCP2* | circadian | 2511 | - | cis-acting regulatory element involved in circadian control |
| *OpTCP2* | ABRE | 2155 | - | cis-acting element involved in the abscisic acid responsiveness |
| *OpTCP2* | ABRE | 2505 | - | cis-acting element involved in the abscisic acid responsiveness |
| *OpTCP2* | ABRE | 2953 | - | cis-acting element involved in the abscisic acid responsiveness |
| *OpTCP2* | CGTCA-motif | 878 | - | cis-acting regulatory element involved in the MeJA-responsiveness |
| *OpTCP2* | CGTCA-motif | 2864 | + | cis-acting regulatory element involved in the MeJA-responsiveness |
| *OpTCP2* | P-box | 2916 | - | gibberellin-responsive element |
| *OpTCP2* | TCA-element | 684 | + | cis-acting element involved in salicylic acid responsiveness |
| *OpTCP2* | TCA-element | 1141 | + | cis-acting element involved in salicylic acid responsiveness |
| *OpTCP2* | TGACG-motif | 878 | + | cis-acting regulatory element involved in the MeJA-responsiveness |
| *OpTCP2* | TGACG-motif | 2864 | - | cis-acting regulatory element involved in the MeJA-responsiveness |
| *OpTCP2* | ARE | 1508 | + | cis-acting regulatory element essential for the anaerobic induction |
| *OpTCP2* | ARE | 2289 | + | cis-acting regulatory element essential for the anaerobic induction |
| *OpTCP2* | ARE | 2300 | - | cis-acting regulatory element essential for the anaerobic induction |
| *OpTCP2* | LTR | 728 | + | cis-acting element involved in low-temperature responsiveness |
| *OpTCP2* | LTR | 896 | + | cis-acting element involved in low-temperature responsiveness |
| *OpTCP2* | MBS | 1810 | + | MYB binding site involved in drought-inducibility |
| *OpTCP2* | WUN-motif | 1308 | - |  |
| *OpTCP3* | O2-site | 970 | + | cis-acting regulatory element involved in zein metabolism regulation |
| *OpTCP3* | O2-site | 2132 | - | cis-acting regulatory element involved in zein metabolism regulation |
| *OpTCP3* | ABRE | 1923 | + | cis-acting element involved in the abscisic acid responsiveness |
| *OpTCP3* | AuxRR-core | 1062 | + | cis-acting regulatory element involved in auxin responsiveness |
| *OpTCP3* | TCA-element | 1771 | + | cis-acting element involved in salicylic acid responsiveness |
| *OpTCP3* | ARE | 2767 | - | cis-acting regulatory element essential for the anaerobic induction |
| *OpTCP3* | MBS | 2199 | + | MYB binding site involved in drought-inducibility |
| *OpTCP3* | MBS | 2207 | + | MYB binding site involved in drought-inducibility |
| *OpTCP3* | MBS | 2213 | + | MYB binding site involved in drought-inducibility |
| *OpTCP3* | MBS | 2221 | + | MYB binding site involved in drought-inducibility |
| *OpTCP3* | WUN-motif | 658 | + |  |
| *OpTCP4* | CAT-box | 2114 | + | cis-acting regulatory element related to meristem expression |
| *OpTCP4* | O2-site | 2593 | - | cis-acting regulatory element involved in zein metabolism regulation |
| *OpTCP4* | circadian | 198 | + | cis-acting regulatory element involved in circadian control |
| *OpTCP4* | ABRE | 1065 | + | cis-acting element involved in the abscisic acid responsiveness |
| *OpTCP4* | ERE | 541 | + |  |
| *OpTCP4* | P-box | 2756 | - | gibberellin-responsive element |
| *OpTCP4* | TCA-element | 2848 | + | cis-acting element involved in salicylic acid responsiveness |
| *OpTCP4* | ARE | 418 | + | cis-acting regulatory element essential for the anaerobic induction |
| *OpTCP4* | ARE | 1068 | - | cis-acting regulatory element essential for the anaerobic induction |
| *OpTCP4* | ARE | 2004 | - | cis-acting regulatory element essential for the anaerobic induction |
| *OpTCP4* | MBS | 696 | + | MYB binding site involved in drought-inducibility |
| *OpTCP4* | MBS | 853 | + | MYB binding site involved in drought-inducibility |
| *OpTCP4* | MBS | 1008 | + | MYB binding site involved in drought-inducibility |
| *OpTCP4* | MBS | 2565 | + | MYB binding site involved in drought-inducibility |
| *OpTCP4* | MBS | 2779 | - | MYB binding site involved in drought-inducibility |
| *OpTCP4* | TC-rich repeats | 2652 | - | cis-acting element involved in defense and stress responsiveness |
| *OpTCP4* | WUN-motif | 1035 | + |  |
| *OpTCP4* | WUN-motif | 1969 | + |  |
| *OpTCP5* | CAT-box | 610 | - | cis-acting regulatory element related to meristem expression |
| *OpTCP5* | CAT-box | 1992 | + | cis-acting regulatory element related to meristem expression |
| *OpTCP5* | CAT-box | 2004 | - | cis-acting regulatory element related to meristem expression |
| *OpTCP5* | O2-site | 1214 | + | cis-acting regulatory element involved in zein metabolism regulation |
| *OpTCP5* | GARE-motif | 2510 | - |  |
| *OpTCP5* | P-box | 1480 | + | gibberellin-responsive element |
| *OpTCP5* | P-box | 2451 | + | gibberellin-responsive element |
| *OpTCP5* | TATC-box | 718 | - | cis-acting element involved in gibberellin-responsiveness |
| *OpTCP5* | TCA-element | 430 | - | cis-acting element involved in salicylic acid responsiveness |
| *OpTCP5* | ARE | 1036 | + | cis-acting regulatory element essential for the anaerobic induction |
| *OpTCP5* | ARE | 1356 | + | cis-acting regulatory element essential for the anaerobic induction |
| *OpTCP5* | ARE | 1454 | + | cis-acting regulatory element essential for the anaerobic induction |
| *OpTCP5* | ARE | 1832 | + | cis-acting regulatory element essential for the anaerobic induction |
| *OpTCP5* | ARE | 2063 | - | cis-acting regulatory element essential for the anaerobic induction |
| *OpTCP5* | ARE | 2411 | - | cis-acting regulatory element essential for the anaerobic induction |
| *OpTCP5* | ARE | 2724 | + | cis-acting regulatory element essential for the anaerobic induction |
| *OpTCP5* | ARE | 2730 | + | cis-acting regulatory element essential for the anaerobic induction |
| *OpTCP5* | ARE | 2742 | + | cis-acting regulatory element essential for the anaerobic induction |
| *OpTCP5* | TC-rich repeats | 78 | + | cis-acting element involved in defense and stress responsiveness |
| *OpTCP5* | TC-rich repeats | 1533 | + | cis-acting element involved in defense and stress responsiveness |
| *OpTCP5* | WUN-motif | 2241 | + |  |
| *OpTCP6* | CAT-box | 185 | - | cis-acting regulatory element related to meristem expression |
| *OpTCP6* | GCN4_motif | 198 | - | cis-regulatory element involved in endosperm expression |
| *OpTCP6* | GCN4_motif | 2895 | - | cis-regulatory element involved in endosperm expression |
| *OpTCP6* | O2-site | 1769 | - | cis-acting regulatory element involved in zein metabolism regulation |
| *OpTCP6* | ABRE | 1146 | + | cis-acting element involved in the abscisic acid responsiveness |
| *OpTCP6* | ABRE | 2610 | - | cis-acting element involved in the abscisic acid responsiveness |
| *OpTCP6* | AuxRR-core | 1346 | + | cis-acting regulatory element involved in auxin responsiveness |
| *OpTCP6* | CGTCA-motif | 173 | + | cis-acting regulatory element involved in the MeJA-responsiveness |
| *OpTCP6* | CGTCA-motif | 2577 | - | cis-acting regulatory element involved in the MeJA-responsiveness |
| *OpTCP6* | ERE | 7 | + |  |
| *OpTCP6* | P-box | 882 | - | gibberellin-responsive element |
| *OpTCP6* | TCA-element | 609 | + | cis-acting element involved in salicylic acid responsiveness |
| *OpTCP6* | TGACG-motif | 173 | - | cis-acting regulatory element involved in the MeJA-responsiveness |
| *OpTCP6* | TGACG-motif | 2577 | + | cis-acting regulatory element involved in the MeJA-responsiveness |
| *OpTCP6* | ARE | 1583 | - | cis-acting regulatory element essential for the anaerobic induction |
| *OpTCP6* | ARE | 2054 | + | cis-acting regulatory element essential for the anaerobic induction |
| *OpTCP6* | MBS | 392 | + | MYB binding site involved in drought-inducibility |
| *OpTCP7* | CAT-box | 520 | - | cis-acting regulatory element related to meristem expression |
| *OpTCP7* | CAT-box | 837 | + | cis-acting regulatory element related to meristem expression |
| *OpTCP7* | GCN4_motif | 1138 | + | cis-regulatory element involved in endosperm expression |
| *OpTCP7* | O2-site | 1111 | - | cis-acting regulatory element involved in zein metabolism regulation |
| *OpTCP7* | O2-site | 2167 | + | cis-acting regulatory element involved in zein metabolism regulation |
| *OpTCP7* | O2-site | 2348 | + | cis-acting regulatory element involved in zein metabolism regulation |
| *OpTCP7* | circadian | 808 | - | cis-acting regulatory element involved in circadian control |
| *OpTCP7* | ABRE | 2221 | - | cis-acting element involved in the abscisic acid responsiveness |
| *OpTCP7* | CGTCA-motif | 612 | - | cis-acting regulatory element involved in the MeJA-responsiveness |
| *OpTCP7* | CGTCA-motif | 1639 | + | cis-acting regulatory element involved in the MeJA-responsiveness |
| *OpTCP7* | TATC-box | 1988 | + | cis-acting element involved in gibberellin-responsiveness |
| *OpTCP7* | TCA-element | 388 | + | cis-acting element involved in salicylic acid responsiveness |
| *OpTCP7* | TCA-element | 746 | + | cis-acting element involved in salicylic acid responsiveness |
| *OpTCP7* | TCA-element | 2093 | + | cis-acting element involved in salicylic acid responsiveness |
| *OpTCP7* | TCA-element | 2819 | + | cis-acting element involved in salicylic acid responsiveness |
| *OpTCP7* | TGACG-motif | 612 | + | cis-acting regulatory element involved in the MeJA-responsiveness |
| *OpTCP7* | TGACG-motif | 1639 | - | cis-acting regulatory element involved in the MeJA-responsiveness |
| *OpTCP7* | ARE | 792 | - | cis-acting regulatory element essential for the anaerobic induction |
| *OpTCP7* | ARE | 1330 | - | cis-acting regulatory element essential for the anaerobic induction |
| *OpTCP7* | ARE | 1916 | - | cis-acting regulatory element essential for the anaerobic induction |
| *OpTCP7* | ARE | 2148 | + | cis-acting regulatory element essential for the anaerobic induction |
| *OpTCP7* | LTR | 224 | - | cis-acting element involved in low-temperature responsiveness |
| *OpTCP7* | MBS | 1066 | + | MYB binding site involved in drought-inducibility |
| *OpTCP7* | MBS | 1143 | - | MYB binding site involved in drought-inducibility |
| *OpTCP7* | TC-rich repeats | 1102 | + | cis-acting element involved in defense and stress responsiveness |
| *OpTCP7* | TC-rich repeats | 2729 | - | cis-acting element involved in defense and stress responsiveness |
| *OpTCP7* | WUN-motif | 732 | + |  |
| *OpTCP7* | WUN-motif | 892 | - |  |
| *OpTCP7* | WUN-motif | 1940 | - |  |
| *OpTCP7* | WUN-motif | 2314 | - |  |
| *OpTCP8* | GCN4_motif | 1597 | - | cis-regulatory element involved in endosperm expression |
| *OpTCP8* | ABRE | 2052 | - | cis-acting element involved in the abscisic acid responsiveness |
| *OpTCP8* | ABRE | 2477 | - | cis-acting element involved in the abscisic acid responsiveness |
| *OpTCP8* | CGTCA-motif | 1045 | - | cis-acting regulatory element involved in the MeJA-responsiveness |
| *OpTCP8* | CGTCA-motif | 2757 | - | cis-acting regulatory element involved in the MeJA-responsiveness |
| *OpTCP8* | ERE | 1771 | - |  |
| *OpTCP8* | TCA-element | 2798 | + | cis-acting element involved in salicylic acid responsiveness |
| *OpTCP8* | TGACG-motif | 1045 | + | cis-acting regulatory element involved in the MeJA-responsiveness |
| *OpTCP8* | TGACG-motif | 2757 | + | cis-acting regulatory element involved in the MeJA-responsiveness |
| *OpTCP8* | ARE | 1662 | + | cis-acting regulatory element essential for the anaerobic induction |
| *OpTCP8* | ARE | 1723 | + | cis-acting regulatory element essential for the anaerobic induction |
| *OpTCP8* | ARE | 2774 | - | cis-acting regulatory element essential for the anaerobic induction |
| *OpTCP8* | LTR | 1232 | + | cis-acting element involved in low-temperature responsiveness |
| *OpTCP8* | LTR | 1406 | - | cis-acting element involved in low-temperature responsiveness |
| *OpTCP8* | LTR | 1625 | - | cis-acting element involved in low-temperature responsiveness |
| *OpTCP8* | MBS | 2532 | + | MYB binding site involved in drought-inducibility |
| *OpTCP8* | TC-rich repeats | 1469 | - | cis-acting element involved in defense and stress responsiveness |
| *OpTCP8* | TC-rich repeats | 1902 | - | cis-acting element involved in defense and stress responsiveness |
| *OpTCP8* | WUN-motif | 1521 | - |  |
| *OpTCP8* | WUN-motif | 2854 | - |  |
| *OpTCP9* | CCGTCC-box | 364 | - |  |
| *OpTCP9* | ABRE | 546 | + | cis-acting element involved in the abscisic acid responsiveness |
| *OpTCP9* | ABRE | 894 | + | cis-acting element involved in the abscisic acid responsiveness |
| *OpTCP9* | ABRE | 1044 | - | cis-acting element involved in the abscisic acid responsiveness |
| *OpTCP9* | AuxRR-core | 1307 | - | cis-acting regulatory element involved in auxin responsiveness |
| *OpTCP9* | CGTCA-motif | 1793 | + | cis-acting regulatory element involved in the MeJA-responsiveness |
| *OpTCP9* | ERE | 329 | + |  |
| *OpTCP9* | TCA-element | 571 | - | cis-acting element involved in salicylic acid responsiveness |
| *OpTCP9* | TGACG-motif | 1793 | - | cis-acting regulatory element involved in the MeJA-responsiveness |
| *OpTCP9* | ARE | 228 | + | cis-acting regulatory element essential for the anaerobic induction |
| *OpTCP9* | LTR | 1124 | + | cis-acting element involved in low-temperature responsiveness |
| *OpTCP9* | MBS | 2682 | + | MYB binding site involved in drought-inducibility |
| *OpTCP9* | WUN-motif | 1590 | - |  |
| *OpTCP10* | CAT-box | 1327 | + | cis-acting regulatory element related to meristem expression |
| *OpTCP10* | HD-Zip 1 | 2766 | - | element involved in differentiation of the palisade mesophyll cells |
| *OpTCP10* | motif I | 1005 | + | cis-acting regulatory element root specific |
| *OpTCP10* | ABRE | 904 | + | cis-acting element involved in the abscisic acid responsiveness |
| *OpTCP10* | ABRE | 1008 | + | cis-acting element involved in the abscisic acid responsiveness |
| *OpTCP10* | ABRE | 1968 | + | cis-acting element involved in the abscisic acid responsiveness |
| *OpTCP10* | ABRE | 2064 | + | cis-acting element involved in the abscisic acid responsiveness |
| *OpTCP10* | ABRE | 2131 | - | cis-acting element involved in the abscisic acid responsiveness |
| *OpTCP10* | ABRE | 2284 | - | cis-acting element involved in the abscisic acid responsiveness |
| *OpTCP10* | CGTCA-motif | 435 | - | cis-acting regulatory element involved in the MeJA-responsiveness |
| *OpTCP10* | CGTCA-motif | 1585 | - | cis-acting regulatory element involved in the MeJA-responsiveness |
| *OpTCP10* | ERE | 1337 | + |  |
| *OpTCP10* | ERE | 1549 | - |  |
| *OpTCP10* | ERE | 2563 | + |  |
| *OpTCP10* | P-box | 611 | + | gibberellin-responsive element |
| *OpTCP10* | P-box | 770 | + | gibberellin-responsive element |
| *OpTCP10* | TCA-element | 2182 | + | cis-acting element involved in salicylic acid responsiveness |
| *OpTCP10* | TGACG-motif | 435 | + | cis-acting regulatory element involved in the MeJA-responsiveness |
| *OpTCP10* | TGACG-motif | 1585 | + | cis-acting regulatory element involved in the MeJA-responsiveness |
| *OpTCP10* | ARE | 1068 | - | cis-acting regulatory element essential for the anaerobic induction |
| *OpTCP10* | ARE | 2466 | + | cis-acting regulatory element essential for the anaerobic induction |
| *OpTCP10* | ARE | 2683 | - | cis-acting regulatory element essential for the anaerobic induction |
| *OpTCP10* | ARE | 2933 | + | cis-acting regulatory element essential for the anaerobic induction |
| *OpTCP10* | WUN-motif | 380 | - |  |
| *OpTCP10* | WUN-motif | 1685 | - | wound-responsive element |
| *OpTCP11* | CAT-box | 1201 | + | cis-acting regulatory element related to meristem expression |
| *OpTCP11* | HD-Zip 1 | 1338 | + | element involved in differentiation of the palisade mesophyll cells |
| *OpTCP11* | O2-site | 1078 | + | cis-acting regulatory element involved in zein metabolism regulation |
| *OpTCP11* | circadian | 1629 | - | cis-acting regulatory element involved in circadian control |
| *OpTCP11* | ABRE | 1237 | - | cis-acting element involved in the abscisic acid responsiveness |
| *OpTCP11* | ABRE | 1705 | + | cis-acting element involved in the abscisic acid responsiveness |
| *OpTCP11* | ABRE | 2484 | + | cis-acting element involved in the abscisic acid responsiveness |
| *OpTCP11* | CGTCA-motif | 398 | + | cis-acting regulatory element involved in the MeJA-responsiveness |
| *OpTCP11* | CGTCA-motif | 659 | - | cis-acting regulatory element involved in the MeJA-responsiveness |
| *OpTCP11* | CGTCA-motif | 937 | - | cis-acting regulatory element involved in the MeJA-responsiveness |
| *OpTCP11* | CGTCA-motif | 1107 | - | cis-acting regulatory element involved in the MeJA-responsiveness |
| *OpTCP11* | ERE | 758 | - |  |
| *OpTCP11* | ERE | 1782 | - |  |
| *OpTCP11* | P-box | 2 | - | gibberellin-responsive element |
| *OpTCP11* | TGACG-motif | 398 | - | cis-acting regulatory element involved in the MeJA-responsiveness |
| *OpTCP11* | TGACG-motif | 659 | + | cis-acting regulatory element involved in the MeJA-responsiveness |
| *OpTCP11* | TGACG-motif | 937 | + | cis-acting regulatory element involved in the MeJA-responsiveness |
| *OpTCP11* | TGACG-motif | 1107 | + | cis-acting regulatory element involved in the MeJA-responsiveness |
| *OpTCP11* | ARE | 226 | + | cis-acting regulatory element essential for the anaerobic induction |
| *OpTCP11* | ARE | 282 | + | cis-acting regulatory element essential for the anaerobic induction |
| *OpTCP11* | ARE | 990 | - | cis-acting regulatory element essential for the anaerobic induction |
| *OpTCP11* | ARE | 1727 | - | cis-acting regulatory element essential for the anaerobic induction |
| *OpTCP11* | ARE | 2296 | + | cis-acting regulatory element essential for the anaerobic induction |
| *OpTCP11* | ARE | 2837 | + | cis-acting regulatory element essential for the anaerobic induction |
| *OpTCP11* | LTR | 2986 | - | cis-acting element involved in low-temperature responsiveness |
| *OpTCP11* | MBS | 1408 | - | MYB binding site involved in drought-inducibility |
| *OpTCP12* | ABRE | 552 | + | cis-acting element involved in the abscisic acid responsiveness |
| *OpTCP12* | ABRE | 740 | + | cis-acting element involved in the abscisic acid responsiveness |
| *OpTCP12* | CGTCA-motif | 2826 | - | cis-acting regulatory element involved in the MeJA-responsiveness |
| *OpTCP12* | GARE-motif | 898 | - |  |
| *OpTCP12* | P-box | 2284 | + | gibberellin-responsive element |
| *OpTCP12* | P-box | 2615 | + | gibberellin-responsive element |
| *OpTCP12* | TGACG-motif | 2826 | + | cis-acting regulatory element involved in the MeJA-responsiveness |
| *OpTCP12* | ARE | 647 | + | cis-acting regulatory element essential for the anaerobic induction |
| *OpTCP12* | ARE | 1854 | + | cis-acting regulatory element essential for the anaerobic induction |
| *OpTCP12* | ARE | 2020 | + | cis-acting regulatory element essential for the anaerobic induction |
| *OpTCP12* | ARE | 2685 | - | cis-acting regulatory element essential for the anaerobic induction |
| *OpTCP12* | MBS | 312 | + | MYB binding site involved in drought-inducibility |
| *OpTCP12* | MBS | 1737 | + | MYB binding site involved in drought-inducibility |
| *OpTCP13* | CAT-box | 2995 | - | cis-acting regulatory element related to meristem expression |
| *OpTCP13* | ABRE | 429 | - | cis-acting element involved in the abscisic acid responsiveness |
| *OpTCP13* | ABRE | 2573 | + | cis-acting element involved in the abscisic acid responsiveness |
| *OpTCP13* | ABRE | 2612 | - | cis-acting element involved in the abscisic acid responsiveness |
| *OpTCP13* | ERE | 518 | - |  |
| *OpTCP13* | ERE | 536 | - |  |
| *OpTCP13* | ERE | 595 | - |  |
| *OpTCP13* | ARE | 2142 | + | cis-acting regulatory element essential for the anaerobic induction |
| *OpTCP13* | ARE | 2218 | - | cis-acting regulatory element essential for the anaerobic induction |
| *OpTCP13* | ARE | 2479 | + | cis-acting regulatory element essential for the anaerobic induction |
| *OpTCP13* | ARE | 2790 | - | cis-acting regulatory element essential for the anaerobic induction |
| *OpTCP13* | LTR | 1334 | + | cis-acting element involved in low-temperature responsiveness |
| *OpTCP13* | MBS | 1474 | + | MYB binding site involved in drought-inducibility |
| *OpTCP13* | WUN-motif | 302 | - |  |
| *OpTCP13* | WUN-motif | 795 | - |  |
| *OpTCP14* | GCN4_motif | 2229 | + | cis-regulatory element involved in endosperm expression |
| *OpTCP14* | ABRE | 110 | - | cis-acting element involved in the abscisic acid responsiveness |
| *OpTCP14* | ABRE | 692 | - | cis-acting element involved in the abscisic acid responsiveness |
| *OpTCP14* | ABRE | 1949 | - | cis-acting element involved in the abscisic acid responsiveness |
| *OpTCP14* | AuxRR-core | 632 | - | cis-acting regulatory element involved in auxin responsiveness |
| *OpTCP14* | CGTCA-motif | 490 | + | cis-acting regulatory element involved in the MeJA-responsiveness |
| *OpTCP14* | ERE | 138 | + |  |
| *OpTCP14* | ERE | 963 | + |  |
| *OpTCP14* | TCA-element | 977 | - | cis-acting element involved in salicylic acid responsiveness |
| *OpTCP14* | TGACG-motif | 490 | - | cis-acting regulatory element involved in the MeJA-responsiveness |
| *OpTCP14* | ARE | 41 | + | cis-acting regulatory element essential for the anaerobic induction |
| *OpTCP14* | ARE | 1785 | + | cis-acting regulatory element essential for the anaerobic induction |
| *OpTCP14* | ARE | 1890 | + | cis-acting regulatory element essential for the anaerobic induction |
| *OpTCP14* | ARE | 1941 | + | cis-acting regulatory element essential for the anaerobic induction |
| *OpTCP14* | ARE | 2096 | - | cis-acting regulatory element essential for the anaerobic induction |
| *OpTCP14* | ARE | 2420 | + | cis-acting regulatory element essential for the anaerobic induction |
| *OpTCP14* | ARE | 2816 | - | cis-acting regulatory element essential for the anaerobic induction |
| *OpTCP14* | MBS | 2092 | - | MYB binding site involved in drought-inducibility |
| *OpTCP14* | TC-rich repeats | 870 | + | cis-acting element involved in defense and stress responsiveness |
| *OpTCP14* | TC-rich repeats | 2695 | - | cis-acting element involved in defense and stress responsiveness |
| *OpTCP14* | WUN-motif | 627 | - |  |
| *OpTCP15* | CAT-box | 2070 | + | cis-acting regulatory element related to meristem expression |
| *OpTCP15* | GCN4_motif | 1913 | + | cis-regulatory element involved in endosperm expression |
| *OpTCP15* | O2-site | 2548 | - | cis-acting regulatory element involved in zein metabolism regulation |
| *OpTCP15* | ABRE | 120 | + | cis-acting element involved in the abscisic acid responsiveness |
| *OpTCP15* | ABRE | 160 | - | cis-acting element involved in the abscisic acid responsiveness |
| *OpTCP15* | ABRE | 869 | + | cis-acting element involved in the abscisic acid responsiveness |
| *OpTCP15* | ABRE | 2271 | - | cis-acting element involved in the abscisic acid responsiveness |
| *OpTCP15* | ABRE | 2549 | - | cis-acting element involved in the abscisic acid responsiveness |
| *OpTCP15* | CGTCA-motif | 1012 | - | cis-acting regulatory element involved in the MeJA-responsiveness |
| *OpTCP15* | CGTCA-motif | 1791 | + | cis-acting regulatory element involved in the MeJA-responsiveness |
| *OpTCP15* | CGTCA-motif | 2551 | + | cis-acting regulatory element involved in the MeJA-responsiveness |
| *OpTCP15* | ERE | 2389 | - |  |
| *OpTCP15* | ERE | 2442 | - |  |
| *OpTCP15* | ERE | 2528 | + |  |
| *OpTCP15* | TCA-element | 2057 | - | cis-acting element involved in salicylic acid responsiveness |
| *OpTCP15* | TGACG-motif | 1012 | + | cis-acting regulatory element involved in the MeJA-responsiveness |
| *OpTCP15* | TGACG-motif | 1791 | - | cis-acting regulatory element involved in the MeJA-responsiveness |
| *OpTCP15* | TGACG-motif | 2551 | - | cis-acting regulatory element involved in the MeJA-responsiveness |
| *OpTCP15* | ARE | 1343 | + | cis-acting regulatory element essential for the anaerobic induction |
| *OpTCP15* | ARE | 2794 | + | cis-acting regulatory element essential for the anaerobic induction |
| *OpTCP15* | MBS | 2917 | - | MYB binding site involved in drought-inducibility |
| *OpTCP15* | WUN-motif | 1081 | + |  |
| *OpTCP16* | CAT-box | 209 | - | cis-acting regulatory element related to meristem expression |
| *OpTCP16* | CAT-box | 1282 | - | cis-acting regulatory element related to meristem expression |
| *OpTCP16* | O2-site | 402 | + | cis-acting regulatory element involved in zein metabolism regulation |
| *OpTCP16* | ABRE | 951 | + | cis-acting element involved in the abscisic acid responsiveness |
| *OpTCP16* | ABRE | 2236 | + | cis-acting element involved in the abscisic acid responsiveness |
| *OpTCP16* | ABRE | 2481 | - | cis-acting element involved in the abscisic acid responsiveness |
| *OpTCP16* | CGTCA-motif | 100 | + | cis-acting regulatory element involved in the MeJA-responsiveness |
| *OpTCP16* | CGTCA-motif | 958 | - | cis-acting regulatory element involved in the MeJA-responsiveness |
| *OpTCP16* | CGTCA-motif | 2336 | + | cis-acting regulatory element involved in the MeJA-responsiveness |
| *OpTCP16* | CGTCA-motif | 2476 | - | cis-acting regulatory element involved in the MeJA-responsiveness |
| *OpTCP16* | ERE | 1321 | - |  |
| *OpTCP16* | ERE | 1444 | + |  |
| *OpTCP16* | P-box | 2686 | + | gibberellin-responsive element |
| *OpTCP16* | TATC-box | 2636 | - | cis-acting element involved in gibberellin-responsiveness |
| *OpTCP16* | TGACG-motif | 100 | - | cis-acting regulatory element involved in the MeJA-responsiveness |
| *OpTCP16* | TGACG-motif | 958 | + | cis-acting regulatory element involved in the MeJA-responsiveness |
| *OpTCP16* | TGACG-motif | 2336 | - | cis-acting regulatory element involved in the MeJA-responsiveness |
| *OpTCP16* | TGACG-motif | 2476 | + | cis-acting regulatory element involved in the MeJA-responsiveness |
| *OpTCP16* | ARE | 2499 | + | cis-acting regulatory element essential for the anaerobic induction |
| *OpTCP16* | LTR | 1717 | - | cis-acting element involved in low-temperature responsiveness |
| *OpTCP16* | TC-rich repeats | 2727 | - | cis-acting element involved in defense and stress responsiveness |
| *OpTCP16* | WUN-motif | 57 | + |  |

Supplementary table 6. Co-expression analysis of OpTCPs and key enzyme genes in CPT biosynthetic pathway in different tissues of *O. pumila*

| **Var1** | **Var2** | **Pathway** | **cor** | **p_value** |
| --- | --- | --- | --- | --- |
| OpTCP3 | CMS | MEP | 0.839846 | 0.004611 |
| OpTCP4 | CMS | MEP | 0.981555 | 2.76E-06 |
| OpTCP4 | DXS | MEP | 0.921145 | 0.000419 |
| OpTCP4 | MECS | MEP | 0.862832 | 0.002745 |
| OpTCP5 | CMS | MEP | 0.955315 | 5.94E-05 |
| OpTCP5 | DXS | MEP | 0.881291 | 0.001687 |
| OpTCP5 | ASA | Shikimate | 0.819444 | 0.006869 |
| OpTCP6 | CMS | MEP | 0.924827 | 0.000356 |
| OpTCP7 | 7-DLH | Iridoid | 0.861182 | 0.002857 |
| OpTCP8 | CMS | MEP | 0.936651 | 0.000198 |
| OpTCP8 | DXS | MEP | 0.887856 | 0.001391 |
| OpTCP8 | MECS | MEP | 0.802104 | 0.009298 |
| OpTCP8 | MK | MVA | 0.817638 | 0.007099 |
| OpTCP8 | ASA | Shikimate | 0.869828 | 0.002302 |
| OpTCP9 | 7-DLH | Iridoid | 0.951012 | 8.17E-05 |
| OpTCP10 | 7-DLH | Iridoid | 0.968202 | 1.83E-05 |
| OpTCP10 | CPR | Iridoid | 0.861364 | 0.002845 |
| OpTCP11 | MK | MVA | 0.879366 | 0.001781 |
| OpTCP11 | ASA | Shikimate | 0.902189 | 0.000875 |
| OpTCP12 | DXR | MEP | -0.837805 | 0.00481 |
| OpTCP13 | CMS | MEP | 0.954067 | 6.54E-05 |
| OpTCP13 | DXS | MEP | 0.87289 | 0.002124 |
| OpTCP13 | MECS | MEP | 0.845615 | 0.004079 |
| OpTCP13 | MK | MVA | 0.856123 | 0.003222 |
| OpTCP14 | DXR | MEP | -0.927823 | 0.00031 |
| OpTCP14 | HDR | MEP | -0.803295 | 0.009115 |
| OpTCP14 | HDS | MEP | -0.883437 | 0.001586 |
| OpTCP14 | IPPI | MEP | -0.844055 | 0.004219 |
| OpTCP14 | AACT | MVA | -0.956259 | 5.52E-05 |
| OpTCP14 | HMGS | MVA | -0.82422 | 0.006285 |
| OpTCP14 | G10H | Iridoid | -0.922361 | 0.000398 |
| OpTCP14 | 7-DLGT | Iridoid | -0.815017 | 0.007442 |
| OpTCP14 | LAMT | Iridoid | -0.820942 | 0.006682 |
| OpTCP15 | CMK | MEP | -0.836683 | 0.004921 |
| OpTCP15 | HDR | MEP | -0.885705 | 0.001484 |
| OpTCP15 | HDS | MEP | -0.847438 | 0.003921 |
| OpTCP15 | IPPI | MEP | -0.844089 | 0.004216 |
| OpTCP15 | 7-DLH | Iridoid | 0.867387 | 0.00245 |
| OpTCP15 | 8-HGO | Iridoid | -0.905702 | 0.000772 |

Supplementary table 7. TBS elements analysis of key enzyme genes involved in CPT biosynthesis

| **Gene name** | **Element** | **Element sequences** | **Start** | **Element length** | **Strand** | **Pathway** |
| --- | --- | --- | --- | --- | --- | --- |
| *OpGPPS* | GGNCC | GGACC | 2909 | 5 | + | Iridoid |
| *OpGPPS* | GGNCC | GGACC | 2942 | 5 | + | Iridoid |
| *OpHMGS* | GGNCC | GGTCC | 339 | 5 | + | MVA |
| *OpHMGS* | GGNCC | GGCCC | 266 | 5 | + | MVA |
| *OpHMGS* | GGNCC | GGGCC | 310 | 5 | + | MVA |
| *OpHMGS* | GCCCR | GCCCA | 1259 | 5 | + | MVA |
| *OpHMGS* | GCCCR | GCCCG | 884 | 5 | - | MVA |
| *OpMDC* | GGNCC | GGCCC | 330 | 5 | + | MVA |
| *OpMDC* | GGNCC | GGGCC | 818 | 5 | + | MVA |
| *OpMDC* | GCCCR | GCCCA | 249 | 5 | + | MVA |
| *OpMDC* | GCCCR | GCCCA | 329 | 5 | + | MVA |
| *OpMDC* | GCCCR | GCCCG | 2219 | 5 | + | MVA |
| *OpSTR* | GGNCC | GGACC | 1319 | 5 | + | DN-CPT |
| *OpSTR* | GGNCC | GGACC | 2425 | 5 | + | DN-CPT |
| *OpSTR* | GGNCC | GGTCC | 1229 | 5 | + | DN-CPT |
| *OpSTR* | GGNCC | GGGCC | 1595 | 5 | + | DN-CPT |
| *OpSTR* | GCCCR | GCCCA | 1164 | 5 | + | DN-CPT |
| *OpSTR* | GCCCR | GCCCA | 1994 | 5 | + | DN-CPT |
| *OpSTR* | GCCCR | GCCCG | 1728 | 5 | + | DN-CPT |
| *OpSTR* | GCCCR | GCCCA | 1596 | 5 | - | DN-CPT |
| *OpSTR* | GCCCR | GCCCA | 2331 | 5 | - | DN-CPT |
| *OpSTR* | GCCCR | GCCCG | 2173 | 5 | - | DN-CPT |
| *Op10HGO* | GGNCC | GGCCC | 171 | 5 | + | Iridoid |
| *Op10HGO* | GGNCC | GGCCC | 1490 | 5 | + | Iridoid |
| *Op10HGO* | GGNCC | GGGCC | 2348 | 5 | + | Iridoid |
| *Op10HGO* | GCCCR | GCCCA | 2173 | 5 | + | Iridoid |
| *Op10HGO* | GCCCR | GCCCA | 525 | 5 | - | Iridoid |
| *Op10HGO* | GCCCR | GCCCA | 1207 | 5 | - | Iridoid |
| *Op10HGO* | GCCCR | GCCCA | 2948 | 5 | - | Iridoid |
| *Op10HGO* | GCCCR | GCCCG | 67 | 5 | - | Iridoid |
| *Op10HGO* | GCCCR | GCCCG | 1489 | 5 | + | Iridoid |
| *Op8HGO* | GGNCC | GGACC | 1076 | 5 | + | Iridoid |
| *Op8HGO* | GGNCC | GGACC | 1469 | 5 | + | Iridoid |
| *Op8HGO* | GGNCC | GGTCC | 1555 | 5 | + | Iridoid |
| *Op8HGO* | GGNCC | GGTCC | 2246 | 5 | + | Iridoid |
| *Op8HGO* | GGNCC | GGTCC | 2756 | 5 | + | Iridoid |
| *Op8HGO* | GGNCC | GGCCC | 1907 | 5 | + | Iridoid |
| *Op8HGO* | GGNCC | GGGCC | 396 | 5 | + | Iridoid |
| *Op8HGO* | GGNCC | GGGCC | 698 | 5 | + | Iridoid |
| *Op8HGO* | GGNCC | GGGCC | 1908 | 5 | + | Iridoid |
| *Op8HGO* | GCCCR | GCCCA | 1906 | 5 | + | Iridoid |
| *Op8HGO* | GCCCR | GCCCA | 1848 | 5 | - | Iridoid |
| *Op8HGO* | GCCCR | GCCCA | 1941 | 5 | - | Iridoid |
| *Op8HGO* | GCCCR | GCCCG | 634 | 5 | - | Iridoid |
| *Op8HGO* | GCCCR | GCCCG | 699 | 5 | - | Iridoid |
| *OpDXR* | GGNCC | GGGCC | 222 | 5 | + | MEP |
| *OpGES* | GCCCR | GCCCA | 1688 | 5 | - | Iridoid |
| *OpGES* | GCCCR | GCCCG | 125 | 5 | + | Iridoid |
| *OpMECS* | GGNCC | GGACC | 1047 | 5 | + | MEP |
| *OpPMK* | GGNCC | GGACC | 1335 | 5 | + | MVA |
| *OpPMK* | GGNCC | GGACC | 2324 | 5 | + | MVA |
| *OpPMK* | GCCCR | GCCCA | 17 | 5 | + | MVA |
| *OpPMK* | GCCCR | GCCCA | 392 | 5 | + | MVA |
| *OpPMK* | GCCCR | GCCCA | 405 | 5 | + | MVA |
| *OpIO* | GGNCC | GGCCC | 1565 | 5 | + | Iridoid |
| *OpIO* | GGNCC | GGCCC | 1571 | 5 | + | Iridoid |
| *OpIO* | GGNCC | GGCCC | 1867 | 5 | + | Iridoid |
| *OpIO* | GCCCR | GCCCA | 1866 | 5 | + | Iridoid |
| *OpIO* | GCCCR | GCCCG | 965 | 5 | + | Iridoid |
| *Op7-DLH* | GGNCC | GGACC | 1188 | 5 | + | Iridoid |
| *Op7-DLH* | GGNCC | GGCCC | 2839 | 5 | + | Iridoid |
| *Op7-DLH* | GCCCR | GCCCA | 729 | 5 | + | Iridoid |
| *Op7-DLH* | GCCCR | GCCCA | 996 | 5 | + | Iridoid |
| *Op7-DLH* | GCCCR | GCCCA | 1111 | 5 | + | Iridoid |
| *Op7-DLH* | GCCCR | GCCCA | 1762 | 5 | + | Iridoid |
| *Op7-DLH* | GCCCR | GCCCA | 2236 | 5 | - | Iridoid |
| *Op7-DLGT* | GGNCC | GGCCC | 1018 | 5 | + | Iridoid |
| *Op7-DLGT* | GGNCC | GGCCC | 2416 | 5 | + | Iridoid |
| *Op7-DLGT* | GGNCC | GGGCC | 1019 | 5 | + | Iridoid |
| *Op7-DLGT* | GGNCC | GGGCC | 1187 | 5 | + | Iridoid |
| *Op7-DLGT* | GGNCC | GGGCC | 2417 | 5 | + | Iridoid |
| *Op7-DLGT* | GCCCR | GCCCA | 1082 | 5 | + | Iridoid |
| *Op7-DLGT* | GCCCR | GCCCA | 2415 | 5 | + | Iridoid |
| *Op7-DLGT* | GCCCR | GCCCA | 1020 | 5 | - | Iridoid |
| *Op7-DLGT* | GCCCR | GCCCA | 2418 | 5 | - | Iridoid |
| *Op7-DLGT* | GCCCR | GCCCA | 2532 | 5 | - | Iridoid |
| *Op7-DLGT* | GCCCR | GCCCG | 1188 | 5 | - | Iridoid |
| *Op7-DLGT* | GCCCR | GCCCG | 2668 | 5 | - | Iridoid |
| *OpSLS* | GGNCC | GGACC | 2264 | 5 | + | Iridoid |
| *OpSLS* | GGNCC | GGCCC | 2158 | 5 | + | Iridoid |
| *OpSLS* | GGNCC | GGCCC | 2319 | 5 | + | Iridoid |
| *OpSLS* | GGNCC | GGGCC | 2389 | 5 | + | Iridoid |
| *OpSLS* | GGNCC | GGGCC | 2455 | 5 | + | Iridoid |
| *OpSLS* | GGNCC | GGGCC | 2562 | 5 | + | Iridoid |
| *OpSLS* | GCCCR | GCCCA | 2157 | 5 | + | Iridoid |
| *OpSLS* | GCCCR | GCCCA | 2212 | 5 | + | Iridoid |
| *OpSLS* | GCCCR | GCCCA | 2318 | 5 | + | Iridoid |
| *OpSLS* | GCCCR | GCCCA | 2348 | 5 | + | Iridoid |
| *OpSLS* | GCCCR | GCCCA | 2382 | 5 | + | Iridoid |
| *OpSLS* | GCCCR | GCCCA | 2399 | 5 | + | Iridoid |
| *OpSLS* | GCCCR | GCCCA | 2448 | 5 | + | Iridoid |
| *OpSLS* | GCCCR | GCCCA | 2148 | 5 | - | Iridoid |
| *OpSLS* | GCCCR | GCCCA | 2326 | 5 | - | Iridoid |
| *OpSLS* | GCCCR | GCCCA | 2390 | 5 | - | Iridoid |
| *OpSLS* | GCCCR | GCCCA | 2456 | 5 | - | Iridoid |
| *OpSLS* | GCCCR | GCCCA | 2563 | 5 | - | Iridoid |
| *OpTDC* | GGNCC | GGACC | 2315 | 5 | + | Shikimate |
| *OpTDC* | GGNCC | GGACC | 1964 | 5 | + | Shikimate |
| *OpTDC* | GGNCC | GGACC | 624 | 5 | + | Shikimate |
| *OpTDC* | GGNCC | GGTCC | 2492 | 5 | + | Shikimate |
| *OpTDC* | GGNCC | GGCCC | 2541 | 5 | + | Shikimate |
| *OpTDC* | GCCCR | GCCCA | 1699 | 5 | + | Shikimate |
| *OpTDC* | GCCCR | GCCCA | 2540 | 5 | + | Shikimate |

Supplementary table 8. Numbers and types of *TCP* genes in higher plants

| Species | Name | Total | I | | II | | | |  |
| --- | --- | --- | --- | --- | --- | --- | --- | --- | --- |
|  |  |  | PCF (num) | PCF (percentage) | CIN (num) | CIN (percentage) | CYC/TB1 (num) | CYC/TB1 (percentage) | |
| *Arabidopsis thaliana* | *AtTCP* | 24 | 13 | 54.2% | 8 | 33.3% | 3 | 12.5% | |
| *Vitis vinifera* | *VvTCP* | 18 | 10 | 55.6% | 5 | 27.8% | 3 | 16.7% | |
| *Oryza sativa* | *OsTCP* | 22 | 10 | 45.5% | 9 | 40.9% | 3 | 13.6% | |
| *Solanum lycopersicum* | *SlTCP* | 30 | 13 | 43.3% | 11 | 36.7% | 6 | 20.0% | |
| *Glycine max* | *GmTCP* | 54 | 26 | 48.1% | 19 | 35.2% | 9 | 16.7% | |
| *Zea mays* | *ZmTCP* | 46 | 17 | 37.0% | 10 | 21.7% | 19 | 41.3% | |
| *Gossypium barbadense* | *GrTCP* | 75 | 50 | 66.7% | 17 | 22.7% | 8 | 10.7% | |
| *Hordeum vulgare* | *HvTCP* | 20 | 10 | 50.0% | 6 | 30.0% | 4 | 20.0% | |
| *Malus domestica* | *MdTCP* | 52 | 22 | 42.3% | 26 | 50.0% | 4 | 7.7% | |
| *Citrullus lanatus* | *ClTCP* | 27 | 12 | 44.4% | 9 | 33.3% | 6 | 22.2% | |
| *Nicotiana tabacum* | *NtTCP* | 61 | 26 | 42.6% | 23 | 37.7% | 12 | 19.7% | |
| *Fragaria vesca* | *FvTCP* | 19 | 10 | 52.6% | 6 | 31.6% | 3 | 15.8% | |
| *Solanum tuberosum* | *StTCP* | 23 | 13 | 56.5% | 7 | 30.4% | 3 | 13.0% | |
| *Phyllostachys edulis* | *PeTCP* | 16 | 10 | 62.5% | 5 | 31.3% | 1 | 6.3% | |
| *Panicum virgatum* | *PvTCP* | 41 | 23 | 56.1% | 12 | 29.3% | 6 | 14.6% | |
| *Populus euphratica* | *PeuTCP* | 33 | 18 | 54.5% | 9 | 27.3% | 6 | 18.2% | |
| *Populus trichocarpa* | *PtTCP* | 36 | 21 | 58.3% | 10 | 27.8% | 5 | 13.9% | |
| *Medicago truncatula* | *MtTCP* | 21 | 9 | 42.9% | 8 | 38.1% | 4 | 19.0% | |
| *Brassica rapa* (Chinese cabbage) | *BrTCP* | 39 | 19 | 48.7% | 14 | 35.9% | 6 | 15.4% | |
| *Ophiorrhiza pumila* | *OpTCP* | 16 | 8 | 50.0% | 6 | 37.5% | 2 | 12.5% | |
